# Excessive branched-chain amino acid accumulation restricts mesenchymal stem cell-based therapy efficacy in myocardial infarction

**DOI:** 10.1038/s41392-022-00971-7

**Published:** 2022-06-03

**Authors:** Fuyang Zhang, Guangyu Hu, Xiyao Chen, Ling Zhang, Lanyan Guo, Congye Li, Hang Zhao, Zhe Cui, Xiong Guo, Fangfang Sun, Dandan Song, Wenjun Yan, Yunlong Xia, Shan Wang, Miaomiao Fan, Ling Tao

**Affiliations:** 1 Department of Cardiology, Xijing Hospital, the Fourth Military Medical University, 710032 Xi’an, China; 2 Department of Geriatrics, Xijing Hospital, the Fourth Military Medical University, 710032 Xi’an, China

**Keywords:** Cardiology, Cardiovascular diseases, Mesenchymal stem cells

## Abstract

Mesenchymal stem cells (MSCs) delivered into the post-ischemic heart milieu have a low survival and retention rate, thus restricting the cardioreparative efficacy of MSC-based therapy. Chronic ischemia results in metabolic reprogramming in the heart, but little is known about how these metabolic changes influence implanted MSCs. Here, we found that excessive branched-chain amino acid (BCAA) accumulation, a metabolic signature seen in the post-ischemic heart, was disadvantageous to the retention and cardioprotection of intramyocardially injected MSCs. Discovery-driven experiments revealed that BCAA at pathological levels sensitized MSCs to stress-induced cell death and premature senescence via accelerating the loss of histone 3 lysine 9 trimethylation (H3K9me3). A novel mTORC1/DUX4/KDM4E axis was identified as the cause of BCAA-induced H3K9me3 loss and adverse phenotype acquisition. Enhancing BCAA catabolic capability in MSCs via genetic/pharmacological approaches greatly improved their adaptation to the high BCAA milieu and strengthened their cardioprotective efficacy. We conclude that aberrant BCAA accumulation is detrimental to implanted MSCs via a previously unknown metabolite-signaling-epigenetic mechanism, emphasizing that the metabolic changes of the post-ischemic heart crucially influence the fate of implanted MSCs and their therapeutic benefits.

## Introduction

Myocardial infarction (MI) compromises cardiac function, remodels the heart structure, and ultimately results in heart failure progression. Stem cell-based therapy is regarded as one of the most promising strategies for ischemic heart injury. In the cardiovascular field, mesenchymal stem cells (MSCs) are the most commonly used cell types in therapy because of their unique properties, including easy isolation and expansion, lack of immunogenicity, no ethical concerns, and multifunctional cardiac repair potential.^1^ Many preclinical investigations with MSCs have been performed and demonstrated the moderate but reproducible benefits of MSC implantation on left ventricular remodeling and heart failure after MI.^2^ However, the cardioreparative efficacy of MSCs remains insufficient, and the salutary effects observed in animal models have not been fully translated to patients suffering from MI. The poor adaptation of implanted MSCs to the stressful post-MI cardiac microenvironment is an unneglected limitation.^3^ As a result, gaining a better understanding of the negative impact of the post-ischemic heart milieu on the retention and cardioprotection of engrafted MSCs is critical for translational progress, and novel strategies are required to overcome this bottleneck that limits MSC implantation’s therapeutic benefits.

Chronic ischemia causes comprehensive reprogramming of myocardial metabolism, resulting in robust changes in intramyocardial metabolite composition and influencing disease progression.^4^ However, whether the metabolic changes in the post-ischemic heart milieu influence the fate of implanted stem cells remains unknown. Branched-chain amino acids (BCAA), consisting of leucine, isoleucine, and valine, constitute a group of essential amino acids that can be highly catabolized in the heart.^5^ We and others have demonstrated that aberrant BCAA accumulation in the post-infarction heart contributes to cardiomyocyte death, pathological ventricular remodeling, and heart failure progression.^6–9^ Recent clinical observations support our original findings, indicating that elevated BCAA levels positively correlate with the risk of adverse cardiovascular events in patients with MI.^10,11^ To date, it remains unclear whether excessive BCAA accumulation in the post-infarct heart influences the retention and cardioprotection of implanted MSCs.

Thus, the primary aim of the present study was to investigate whether aberrant BCAA accumulation may adversely regulate the fate of implanted MSCs in the post-ischemic heart. Upon revealing the impact of excessive BCAA accumulation on the fate of implanted MSCs, we aimed to discover the underlying mechanisms via bioinformatics analyses and experimental approaches and, accordingly, develop novel strategies to improve the cardioprotective efficacy of MSC-based therapy in ischemic heart injury. In the present study, we have for the first time provided evidence that excessive BCAA accumulation adversely influences the survival, retention, and cardioprotective efficacy of implanted MSCs. Mechanistically, BCAA at pathological concentrations drove MSCs vulnerable to detrimental stress-induced cell death and premature senescence via a novel metabolite-signaling-epigenetic axis. The study provides new insights, from a metabolic point of view, on improving the therapeutic efficacy of MSC-based therapy in ischemic heart injury.

## Results

### Excessive BCAA accumulation was disadvantageous to implanted ADSC retention and cardioprotection in the post-MI heart

To characterize the metabolic alterations in the post-infarcted heart, we examined cardiac transcriptome data of human and mouse ischemic cardiomyopathy available from the Gene Expression Omnibus (GEO) database (GSE48166 and GSE95755). The valine, leucine, and isoleucine degradation pathway (KO00280) was one of the most substantially downregulated metabolic pathways in the hearts of both patients and animals with ischemic cardiomyopathy, according to the Kyoto Encyclopaedia Genes and Genomes (KEGG) analysis (supplementary Fig. 1a, b). These results indicated that defective BCAA catabolism represents a metabolic hallmark of the ischemic heart. Given these observations, we focused on whether excessive BCAA accumulation in the post-ischemic heart influences the fate of implanted MSCs.

Adipose-derived mesenchymal stem cells (ADSCs) were isolated from Sprague–Dawley rats, and their purity and pluripotency were validated in experiments that we have previously described.^12^ To determine whether defective BCAA catabolism and subsequent BCAA accumulation in the post-infarction heart affects the retention and cardioprotection of implanted MSCs, ADSCs (passage 2, 2 × 10^5^ cells) were intramyocardially injected immediately after MI into the peri-infarction myocardial tissues of the mitochondrial matrix-targeted protein phosphatase 2C family member (PP2Cm) knockout (KO) mice or their wild-type (WT) littermates. PP2Cm is the phosphatase essential for the dephosphorylation (activation) of branched-chain keto acid dehydrogenase E1 subunit α (BCKDHA), the rate-limiting enzyme in BCAA catabolism.^13^ Thus, PP2Cm KO mice have been widely used as appropriate rodent models for BCAA catabolic abnormalities.^7,14^ The phosphorylation (inactivation) of serine 293 in BCKDHA was dramatically elevated in the PP2Cm-KO hearts, as determined by western blot analysis (supplementary Fig. 1c). In line with our prior findings, there was a significant accumulation of BCAA in the WT heart after MI (supplementary Fig. 1d). During the observation period following MI, intramyocardial BCAA concentrations in the PP2Cm KO hearts were significantly higher than in the WT hearts (supplementary Fig. 1d).

**Fig. 1:**
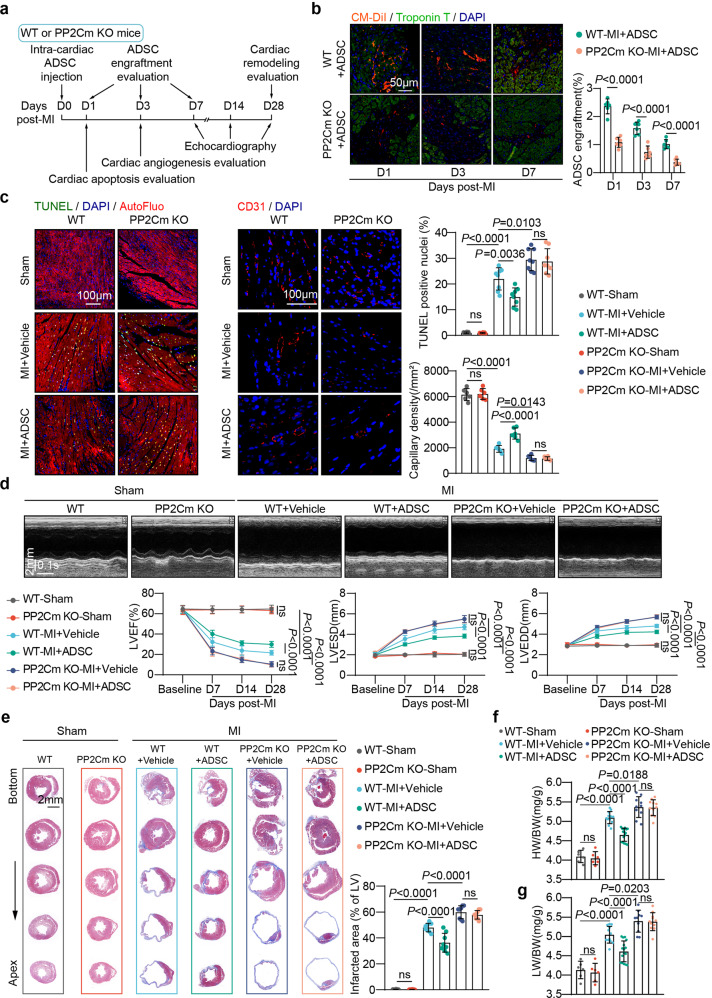
Excessive BCAA accumulation in the post-infarction heart was disadvantageous for implanted ADSC retention and cardioprotection. **a** Experimental scheme using WT or PP2Cm KO mice subjected to intra-cardiac ADSC injection following MI. **b** Left panel, representative images of CM-DiI-labeled ADSCs (red) in the peri-infarct region 1, 3, and 7 d post-MI. Troponin T staining (green) indicates cardiomyocytes. Right panel, number of engrafted ADSCs normalized to the number of cardiomyocytes. (**c**) Left panel, representative images of TUNEL stained cells (left) and CD31 immunostaining (middle) in the peri-infarction zone 3-d post-MI. Right panel, quantification of TUNEL and CD31 staining intensity. **d** Top panel, representative echocardiographic images taken 28 d post-MI. Bottom panel, LVEF, LVESD, and LVEDD values at baseline and 7, 14, and 28 d after MI. *n* = 6–16. **e** Left panel, representative images of Masson’s trichrome staining from the bottom to the apex of hearts 28 d post-MI. Right panel, quantification values of infarcted area. **f** HW/BW ratios 28 d post-MI. **g** LW/BW ratios 28 d post-MI The data are shown as the means ± SD. The engraftment rates of the ADSCs as determined by unpaired Student’s *t* tests. Other data were analysed by two-way ANOVAs with repeated measures followed by Bonferroni post hoc test. ADSCs adipose-derived mesenchymal stem cells, AutoFluo autofluorescence, BCAA branched chain amino acids, HW/BW heart weight/body weight ratio, LW/BW lung weight/body weight ratio, LVEF left ventricular ejection fraction, LVESD left ventricular end-systolic dimension, LVEDD left ventricular end diastolic dimension, MI myocardial infarction, PP2Cm KO mitochondrial matrix-targeted protein phosphatase 2C family member knockout, TUNEL transferase-mediated dUTP nick-end labeling, WT wild type

The retention rate of the ADSCs labeled by the living cell tracker CM-DiI was assessed on day 1, 3, and 7 after MI, a period during which ADSCs were gradually lost in the post-infarction heart. Notably, the engraftment rate of ADSCs injected into the PP2Cm KO heart was much lower than in the WT heart (Fig. 1b). Intramyocardial delivery of ADSCs reduced post-MI cardiomyocyte apoptosis and enhanced angiogenesis in WT animals, as expected. When injected into the PP2Cm KO hearts, however, these beneficial effects of ADSCs were entirely lost (Fig. 1c). Consecutive echocardiography showed that intramyocardial ADSC treatment improved post-MI ventricular dysfunction and enlargement when delivered in the WT group, as evidenced by increased left ventricular ejection fractions (LVEF) and decreased left ventricular end-systolic (LVESD) and end-diastolic diameters (LVEDD) (Fig. 1d). These salutary effects were eliminated when ADSCs were injected into the PP2Cm KO hearts after MI (Fig. 1d). Masson’s trichrome staining revealed that ADSCs ameliorated ventricular enlargement and fibrotic scar formation after MI (Fig. 1e). However, when injected into the PP2Cm KO hearts characterized by excessive BCAA accumulation, these above-mentioned protective benefits were not observed (Fig. 1e). ADSC therapy reduced the heart weight/body weight ratio (HW/BW) and lung weight/body weight ratio (LW/BW) in the WT group following MI (Fig. 1f, g), indicating that ADSC therapy reduces cardiac hypertrophy and lung congestion. When delivered into the post-MI PP2Cm-KO hearts, these benefits mediated by ADSCs were not evident (Fig. 1f, g).

Another series of experiments were performed to evaluate the impact of aberrant BCAA accumulation on the retention and cardioprotection of implanted MSCs in the post-infarction heart. To intervene cardiac BCAA levels, the mice that received intra-cardiac ADSC delivery were daily fed a BCAA mixture in drinking water for 7 consecutive days after MI (supplementary Fig. 2a). Consistent with our previous findings, cardiac BCAA levels were significantly higher in the post-infarction hearts of the mice fed BCAA compared to the vehicle group (supplementary Fig. 2b).^6,7^ Intriguingly, dietary BCAA intervention significantly reduced the retention of ADSCs in the post-infarction heart, as observed in the PP2Cm KO models (supplementary Fig. 2c). As a result, the benefits of ADSC therapy on cardiac dysfunction and structural remodeling were greatly diminished by oral BCAA administration (supplementary Fig. 2d–g). Combined with the results observed in PP2Cm KO mice, these findings collectively indicate that excessive BCAA accumulation, no matter caused by genetic or dietary intervention, is disadvantageous for the engraftment and cardioprotection of ADSCs when intramyocardially delivered into the post-MI heart.

### BCAA at pathological levels sensitized ADSCs to stress-induced premature senescence and death

**Fig. 2:**
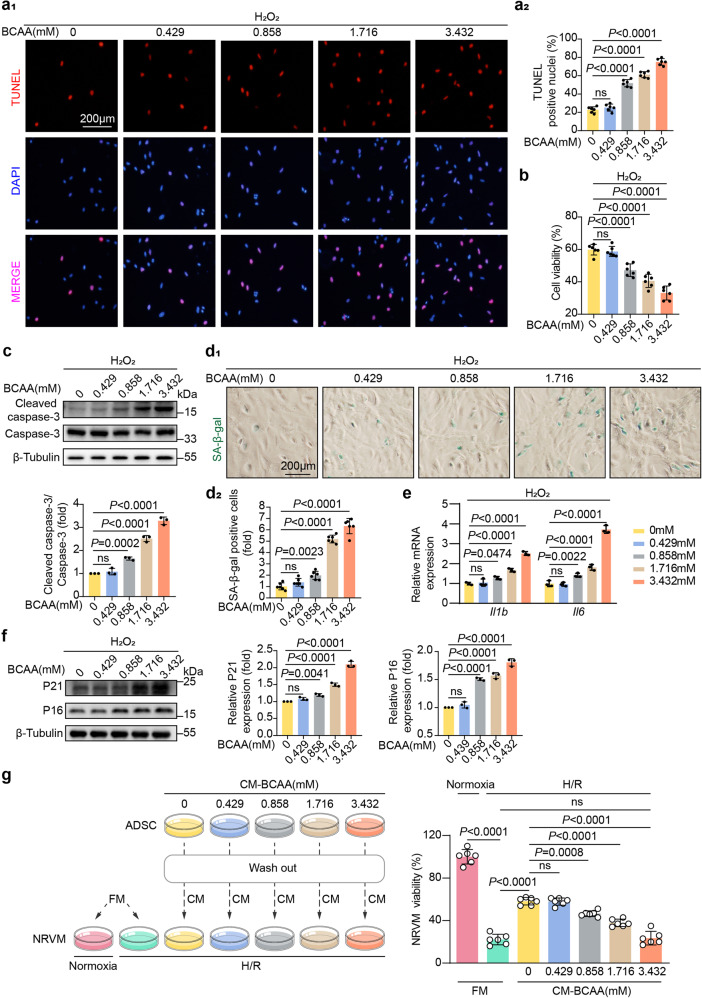
BCAA sensitized ADSCs to detrimental stress-induced death and premature senescence. **a** a1 panel, representative images of TUNEL staining. ADSCs were incubated with increasing doses of BCAA for 24 h with hydrogen peroxide (100 μM)-induced stress. a2 panel, quantification of TUNEL staining intensity. **b** Viability of ADSCs as determined by CCK-8 assay. **c** Top panel, representative blots showing cleaved caspase-3, caspase-3, and β-tubulin levels. Bottom panel, ratio of quantified cleaved caspase-3 expression to caspase-3 expression. **d** d1, representative images of SA-β-gal stained cells. ADSCs were incubated with the indicated BCAA under hydrogen peroxide (100 μM)-induced premature senescence as methods described. d2, quantification of SA-β-gal-positive cells. **e** mRNA levels of Il1b and Il6 as measured by RT-qPCR and normalized to the level of *Actb* mRNA. **f** Representative western blot images and quantification values of P21 and P16. The expression of β-tubulin was used as the loading control. **g** Left panel, schematic diagram showing ADSCs exposed to hydrogen peroxide (100 μM) and treated with increasing doses of BCAA for 24 h. Twelve hours after the ADSCs were washed, fresh or conditioned medium was added to NRVMs subjected to normoxia or H/R. Right panel, NRVM viability was determined by CCK-8 assay. The data are shown as the means ± SD. The data were analysed by one-way ANOVA, followed by Bonferroni post hoc test. *Actb* β-actin, ADSCs adipose tissue-derived mesenchymal stem cells, BCAA branched chain amino acids, CCK-8 Cell Count Kit-8, CM conditioned medium, FM fresh medium, H/R hypoxia/reoxygenation, Il1b interleukin-1β, Il6 interleukin-6, NRVMs neonatal rat ventricular myocytes, SA-β-gal senescence-associated β-galactosidase, TUNEL transferase-mediated dUTP nick-end labeling

We next evaluated the direct impact of BCAA on the injured phenotypes of ADSCs upon physiological or stressed conditions. Under physiological conditions, increasing doses of BCAA ranging from 0 to 0.432 mM (physiological concentration in the circulation) and from 0.858 to 3.432 mM (pathological concentrations seen under diseased conditions) were added into cultured ADSCs.^7–9^ No remarkable cellular apoptosis (as determined by cell viability assay and cleaved caspase-3 expression level) or premature senescence (as determined by the acquisition of senescence-associated secretory phenotypes and β-galactosidase staining) was observed in the ADSCs treated with different doses of BCAA (supplementary Fig. 3a–d). These results demonstrate that, as essential nutrients, BCAA are nontoxic to ADSCs in physiological conditions. Detrimental stress is often seen in the post-infarction heart, such as oxidative stress, is experimentally mimicked by hydrogen peroxide treatment, and intriguingly, when compared to the BCAA-free (0 mM) group, BCAA administered at physiological concentrations (0.432 mM) in the presence of hydrogen peroxide did not exacerbate ADSC apoptosis, as determined by TUNEL staining, cell viability assay, and cleaved caspase-3 expression level (Fig. 2a–c). However, BCAA administered at pathological concentrations (ranging from 0.858 to 3.432 mM) increased the apoptotic rate of ADSCs treated with hydrogen peroxide in a dose-dependent manner (Fig. 2a–c). These results demonstrate that BCAA at pathological concentrations sensitize ADSCs to external detrimental stress, causing cellular death. External stimuli, such as oxidative stress, generate a premature senescent phenotype in MSCs, making them more vulnerable to cell death and limiting their tissue reparative potential.^15^ When exposed to physiological levels of BCAA (0.432 mM), hydrogen peroxide-induced senescent phenotypes were not changed in the ADSCs (Fig. 2d–f). However, when treated with BCAA at pathological concentrations ranging from 0.858 to 3.432 mM, the premature senescent phenotype acquisition of ADSCs induced by hydrogen dioxide was gradually exacerbated as the concentrations of BCAA increased, as shown by an increase in senescence-associated β-galactosidase (SA-β-gal)-positive cells and higher expression levels of interleukin-1b (*Il1b*) and interleukin-6 (*Il6*), two genes related to the senescence-associated secreted phenotype (SASP) (Fig. 2d, e). BCAA at pathological concentrations also increased the expression of p16 and p21, two molecular markers of cellular senescence. (Fig. 2f). Considering the observations that BCAA at pathological levels sensitized ADSCs to oxidative stress-induced apoptosis and premature senescence, we questioned whether BCAA influence the cardioreparative potential of ADSCs. As expected, conditioned medium (CM) collected from ADSCs cultures significantly ameliorated hypoxia/reoxygenation (H/R)-induced neonatal rat ventricular myocyte (NRVM) death, indicating that ADSCs have paracrine cardioprotective potential (Fig. 2g). Notably, the protective paracrine effect of the ADSCs was significantly attenuated upon exposure to pathological concentrations of BCAA (Fig. 2g). Collectively, these in vitro results demonstrate that BCAA at pathological levels sensitize ADSCs to external stress-induced death and premature senescent phenotype acquisition, thereby suppressing their paracrine cardioprotective potential.

### BCAA at pathological levels accelerated H3K9me3 loss in ADSCs

**Fig. 3:**
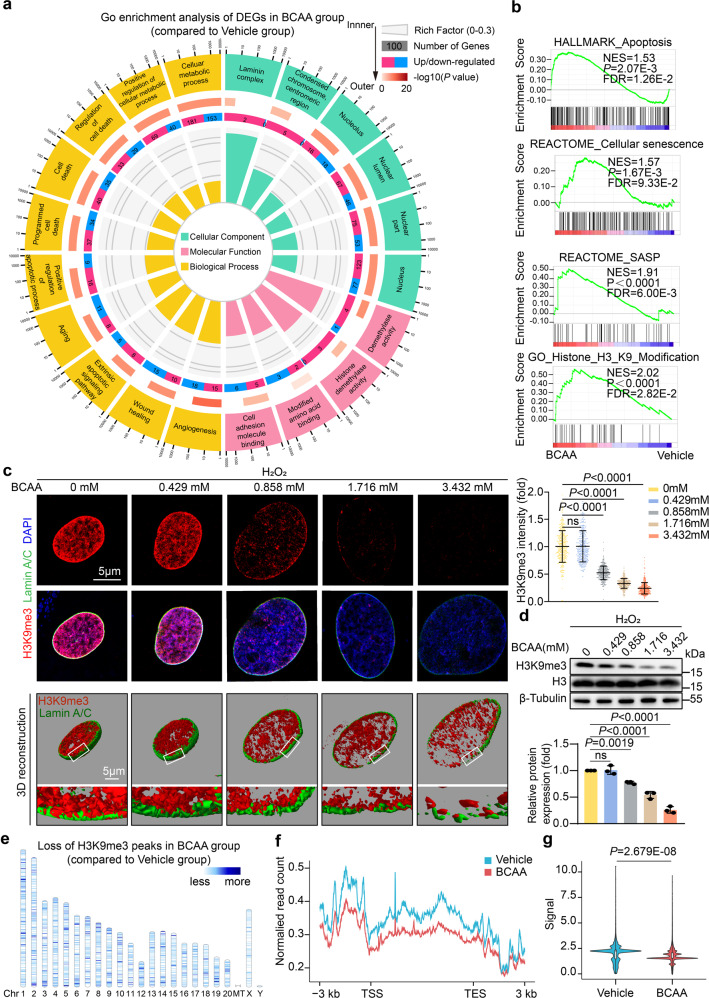
BCAA accelerated H3K9me3 loss in ADSCs upon exposure to detrimental stress. **a** GO enrichment of the DEGs in ADSCs treated with vehicle or BCAA (3.432 mM) under hydrogen peroxide (100 μM) stress as identified by RNA-seq. Twenty significantly enriched GO terms are shown. **b** GSEA showed that apoptosis, cellular senescence, SASP, and H3K9 modification were enriched in ADSCs treated with BCAA. **c** Left panel, representative images of stained H3K9me3 (red) and lamin A/C (green) in ADSCs incubated with increasing doses of BCAA under hydrogen peroxide (100 μM) induced premature senescence as methods described. Three-dimensional Z-stack reconstruction of immunostaining images is shown at the bottom. Right panel, quantification of stained H3K9me3 intensity as determined by ImageJ software. n = 300 per group. **d** Top panel, representative blots of H3K9me3, H3 histone, and β-tubulin. β-Tubulin was used as the loading control. Bottom panel, quantification of H3K9me3 normalized to that of H3. **e** CUT&Tag analysis showed the loss of H3K9me3 across 23 chromosomes in ADSCs treated with BCAA (3.432 mM) compared to the vehicle cells. The scale means the signal loss of H3K9me3 modification in the genome sites. **f** Line plot showing the loss of H3K9me3 signals from 3 kb upstream of the TSS to 3 kb downstream of the TES across the genome in ADSCs treated with BCAA (3.432 mM) compared to the vehicle cells. **g** Violin plot showing the significant decrease in H3K9me3 signals across the whole genome in ADSCs treated with BCAA compared to the vehicle cells. The data are shown as the means ± SD. The data presented in **c** and **d** were analysed by one-way ANOVA followed by a Bonferroni post hoc test. The data shown in **g** were analysed by unpaired Student’s *t* test. ADSCs adipose tissue-derived mesenchymal stem cells, BCAA branched-chain amino acids, CUT&Tag cleavage under targets and tagmentation, DEG differentially expressed genes, GSEA gene set enrichment analysis, GO gene ontology, H3K9me3 histone H3K9 trimethylation, SASP senescence-associated secreted phenotypes, TSS transcriptional start site, TES transcriptional end site

To discover the mechanisms underlying the detrimental effects of BCAA, we performed RNA sequencing (RNA-Seq) with ADSCs treated with vehicle or BCAA at pathological concentrations (3.432 mM) upon oxidative stress. In line with the observations indicating that BCAA sensitized ADSCs to cell death and senescence, the gene set enrichment analysis (GSEA) showed that the pathways related to apoptosis, senescence, and the SASP were significantly enriched in BCAA group (Fig. 3a, b). Surprisingly, Gene Ontology (GO) enrichment analysis revealed that differentially expressed genes (DEGs) in BCAA-treated ADSCs were enriched in the cellular component terms including nucleus, laminin complex, condensed chromosome, and in the molecular function term histone demethylase activity, providing a previously unrecognized indication that BCAA might cause epigenetic alterations in ADSCs (Fig. 3a, b). Notably, histone 3 lysine 9 (H3K9) modification is one of the most significant epigenetic terms enriched in BCAA-treated ADSC DEGs (Fig. 3b). The loss of histone H3K9 trimethylation (H3K9me3), a histone modification required for the maintenance of heterochromatin, is recently characterized as an epigenetic characteristic of ageing MSCs.^16^ Moreover, stabilizing heterochromatin by maintaining H3K9me3 abundance rejuvenates senescent MSCs and mobilizes their tissue-reparative potential.^16,17^ Thus, we further observed the impact of BCAA on H3K9me3 in ADSCs. Without any external stress, BCAA exerted little impact on H3K9me3 abundance in ADSCs (supplementary Fig. 4a). However, in response to oxidative stress mimicked by hydrogen peroxide, BCAA at pathological levels markedly accelerated the loss of H3K9me3 in ADSCs, as determined by immunostaining and western blot analysis (Fig. 3c, d). In contrast, the levels of other common histone H3 modifications, such as H3K4me3 and H3K27me3, remained unchanged in BCAA-treated ADSCs exposed to hydrogen peroxide challenge (supplementary Fig. 4b).

H3K9me3 is essential for the formation of heterochromatin and the maintenance of nuclear integrity. In addition to inducing a decrease in H3K9me3, BCAA at pathological levels resulted in the enlargement of the nucleus and an increase in chromatin heterogeneity, a manifestation of heterochromatin loss (supplementary Fig. 5). In addition, 3-dimensional reconstruction showed that the loss of H3K9me3 was accompanied by nuclear envelope discontinuity in BCAA-challenged ADSCs (Fig. 3c). Cleavage under targets and tagmentation (CUT&Tag) assays were performed to measure the direct interaction between H3K9me3 and genomic DNA. In comparison to the vehicle group, a genome-wide loss of H3K9me3 occupancy across the transcription start site and transcription end site on 23 chromosomes was observed in BCAA-treated ADSCs (Fig. 3e–g). Taken together, the results demonstrate that BCAA accelerate the loss of H3K9me3, heterochromatin, and nuclear integrity in ADSCs upon oxidative stress.

### KDM4E upregulation was responsible for H3K9me3 loss and adverse phenotype acquisition of ADSCs induced by BCAA

**Fig. 4:**
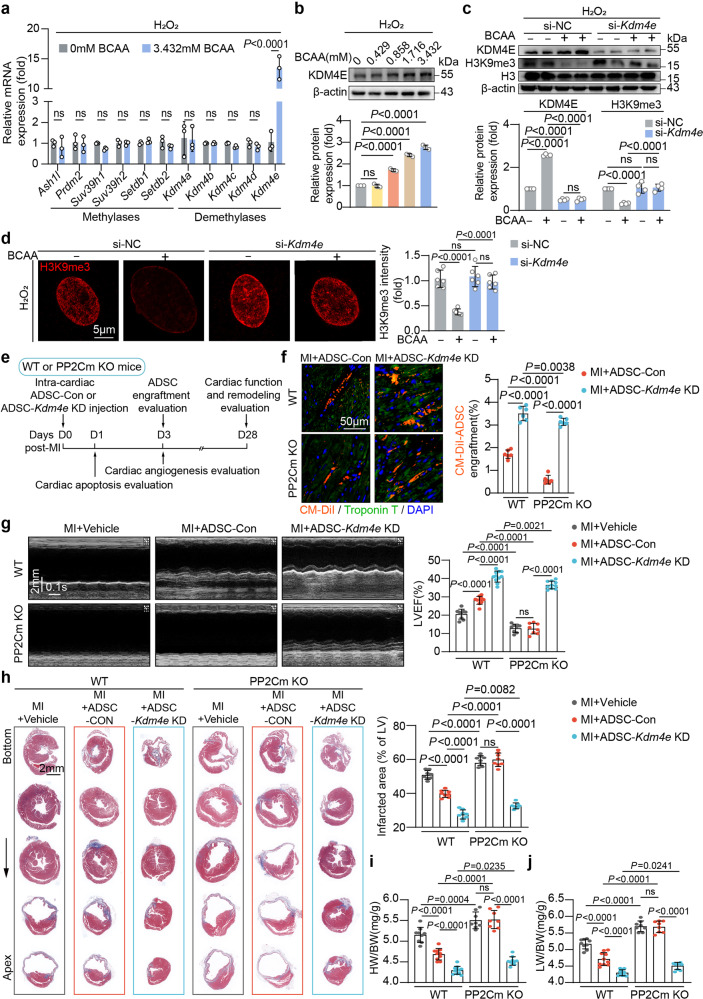
KDM4E was responsible for BCAA-associated H3K9me3 loss, adverse phenotype acquisition, and cardioprotection impairment in ADSCs. **a** mRNA levels of H3K9-specific methylases and demethylases in ADSCs treated with or without BCAA (3.432 mM) in the presence of hydrogen peroxide (100 μM) for 24 h. *Actb* was used as the endogenous control gene. **b** Representative western blots and quantification of KDM4E in ADSCs treated with different doses of BCAA under hydrogen peroxide (100 μM)-induced premature senescence as methods described. β-Actin was used as the loading control. **c** Representative blots and quantification of KDM4E, H3K9me3, H3, and β-actin in ADSCs transfected with scramble (si-NC) or *Kdm4e* siRNA (si-*Kdm4e*) and treated with or without BCAA (3.432 mM) under hydrogen peroxide (100 μM)-induced premature senescence as methods described. **d** Representative image and quantification of H3K9me3 intensity in ADSCs transfected with si-NC or si-*Kdm4e* and treated with or without BCAA (3.432 mM) under hydrogen peroxide (100 μM)-induced premature senescence as methods described. **e** Schematic illustration of the experiment. ADSCs labeled by the live-cell tracker CM-DiI were transfected with scramble (ADSC-Con) or siRNA specific for Kdm4E (ADSC-*Kdm4e* KD), and were intramyocardially injected into the WT or PP2Cm KO mice immediately after MI. **f** Left panel, representative images of CM-DiI-labeled ADSCs (red) in the peri-infarct region 3-d post-MI. Troponin T staining (green) indicates cardiomyocytes. Right panel, number of engrafted ADSCs normalized to the number of cardiomyocytes. **g** Representative echocardiographic images taken 28 d post-MI and LVEF were calculated. **h** Left panel, representative images of Masson’s trichrome staining from the bottom to the apex of hearts 28 d post-MI. Right panel, quantification values of infarcted area. The data are shown as the means ± SD. **i** HW/BW ratios 28 d post-MI. **j** LW/BW ratios 28 d post-MI. The data are shown as the means ± SD. The data shown in **a** and **f** were analysed by unpaired Student’s *t* test. The data shown in **b**, **g**, **h**, **i**, and **j** was analysed by one-way ANOVA followed by Bonferroni post hoc test. The data shown in **c** and **d** were analysed by two-way ANOVA followed by a Bonferroni post hoc test. ADSCs adipose tissue-derived mesenchymal stem cells, BCAA branched chain amino acids, DUX4 double homeobox protein 4, HW/BW heart weight/body weight, H3K9me3 histone H3K9 trimethylation, KDM4E lysine-specific demethylase 4E, KO knockout, LW/BW lung weight/body weight, PP2Cm mitochondrial matrix-targeted protein phosphatase 2C family member, WT wild type

H3K9me3 is controlled by the balance between the methylases and demethylases. To determine how BCAA trigger the loss of H3K9me3 in ADSCs, we examined the expression levels of histone methylases and demethylases specific for H3K9me3 in vehicle- or BCAA-treated ADSCs. The results showed that, among the candidates, only lysine-specific demethylase 4E (KDM4E) mRNA was increased, by approximately 15-fold, in response to BCAA treatment (Fig. 4a). A western blot analysis revealed that BCAA at pathological concentrations upregulated the protein expression of KDM4E in ADSCs in a dose-dependent fashion (Fig. 4b). Silencing of KDM4E by small interfering RNA (siRNA) reversed the decrease in H3K9me3 in BCAA-treated ADSCs, as determined by both immunostaining and western blot analysis (Fig. 4c, d), suggesting that KDM4E plays an indispensable role in BCAA-induced H3K9me3 loss. Notably, the exacerbation of hydrogen peroxide-induced cell death and senescence in BCAA-treated ADSCs was completely reversed by KDM4E silencing (supplementary Fig. 6a–c). These results demonstrate that BCAA sensitize ADSCs to cell death and stress-induced premature senescence via KDM4E-mediated H3K9me3 loss.

### KDM4E-mediated H3K9me3 loss was responsible for the inadaptation of ADSCs to the high BCAA levels in the post-infarcted heart

An important point is to determine whether KDM4E-mediated H3K9me3 loss influences the adaptation and cardioprotection of ADSCs implanted into the high BCAA milieu seen in the post-infarction heart. Following MI, ADSCs transfected with scramble RNA (ADSC-Con) or KDM4E siRNA (ADSC-*Kdm4e* KD) were delivered intramyocardially into WT or PP2Cm KO hearts (Fig. 4e). The ADSCs were labeled by the cell tracker CM-DiI. Consistent with the observation in Fig. 1, the loss of ADSC-Con was significantly accelerated when delivered into the PP2Cm KO heart characterized by overwhelming BCAA accumulation (Fig. 4f). In contrast, silencing KDM4E in ADSCs increased their engraftment rate after MI regardless of whether they were delivered into the WT or PP2Cm KO heart (Fig. 4f). These results provide in vivo evidence that excessive BCAA accumulation results in the inadaptation of MSCs to the post-infarcted heart milieu via inducing KDM4E expression. As a consequence, the pro-survival and pro-angiogenetic effects of ADSC-Con were greatly weakened when delivered into the PP2Cm KO heart (supplementary Fig. 6d, e). The cardioprotective effects mediated by ADSC-Con seen in the WT heart were eliminated in the PP2Cm KO heart (Fig. 4g–j). In both WT and PP2Cm KO hearts, silencing of KDM4E markedly improved the therapeutic potential of ADSCs on cardiac dysfunction and remodeling after MI to the same degree (supplementary Fig. 6d, e and Fig. 4g–j), demonstrating in vivo that KDM4E silencing overcomes the adverse effects on the adaptation and cardioprotection mediated by the high BCAA levels in the post-infarction heart.

### BCAA-mTORC1-DUX4 constituted a novel axis regulating KDM4E transcription in ADSCs

**Fig. 5:**
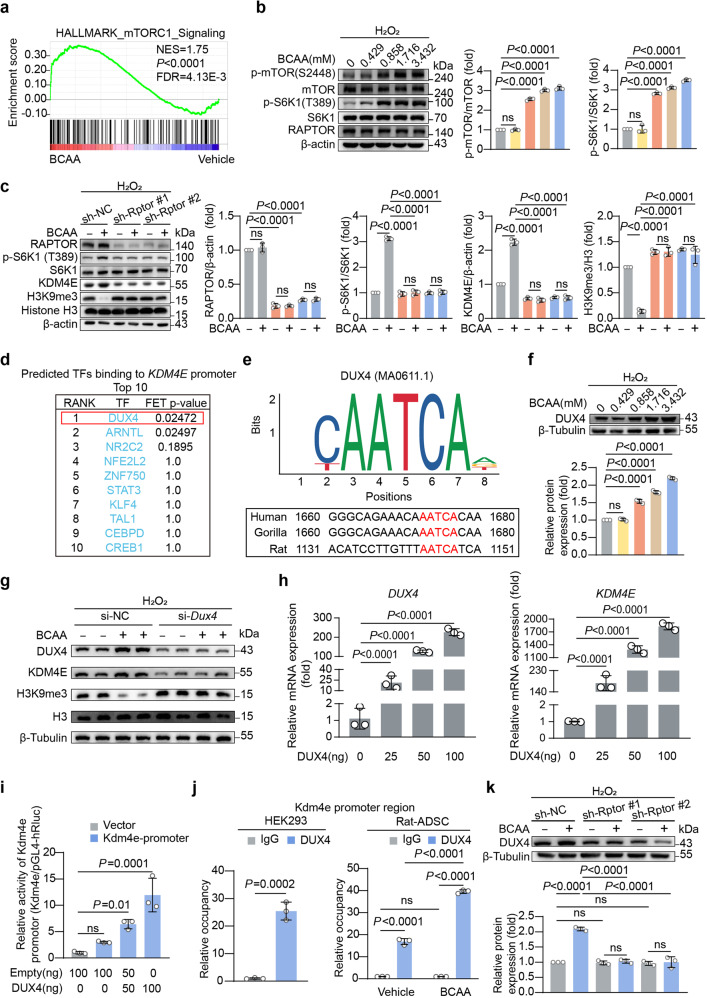
A novel mTORC1/DUX4/KDM4E axis was critical for BCAA-mediated H3K9me3 loss in ADSCs. **a** GSEA showed that the mTORC1 signaling pathway was significantly enriched in BCAA-treated ADSCs. **b** Representative blots of p-mTOR (S2448), mTOR, p-S6K1 (T389), S6K1, RAPTOR, and β-actin protein in ADSCs treated with increasing doses of BCAA in the presence of hydrogen peroxide (100 μM). **c** ADSCs were transfected with sh-*Rptor*#1, sh-*Rptor*#2, or sh-NC and treated with or without BCAA (3.432 mM) in the presence of hydrogen peroxide (100 μM). Representative blots of RAPTOR, p-S6K1 (T398), S6K1, KDM4E, H3K9me3, histone H3, and β-actin are shown. **d** Predicted TFs binding to the *Kdm4e* gene promoter region. The promoter region was defined as the 2000 bp upstream of the transcription start site of the *Kdm4e* gene. **e** Binding motif of DUX4 and its predicted binding site in the *Kdm4e* gene promoter region. **f** Representative blots and quantification of DUX4 protein in ADSCs treated with different doses of BCAA in the presence of hydrogen peroxide (100 μM). **g** ADSCs were transfected with si-NC or si-*Dux4* and treated with or without BCAA (3.432 mM) in the presence of hydrogen peroxide (100 μM). Representative blots of DUX4, KDM4E, H3K9me3, H3, and β-tubulin are shown. **h** mRNA levels of *Dux4* and *Kdm4e* in HEK293 cells transfected with plasmids overexpressing *Dux4*. *Actb* was used as the endogenous control gene. **i** Dual-luciferase activity assay. Vectors overexpressing *Dux4* or empty vectors were co-transfected with the firefly luciferase reporter driven by the *Kdm4e* promoter in HEK293 cells. Renilla luciferase activity was used as a transfection control. **j** Enrichment of DUX4 protein within the promoter regions of the *Kdm4e* gene in HEK293 cells and ADSCs as determined by ChIP-qPCR. **k** Representative blots and quantification of DUX4 protein in sh-NC-, sh-*Rptor*#1-, and sh-*Rptor*#2-transfected ADSCs with or without BCAA treatment (3.432 mM) for 24 h. The data are shown as the means ± SD. The data shown in **b**, **e**, **g**, **i**, and **j** were analysed by one-way ANOVA followed by Bonferroni post hoc test. The data shown in **c**, **g**, and **k** were analysed by two-way ANOVA followed by a Bonferroni post hoc test. The data shown in **j** was analysed by unpaired Student’s *t* test. ADSCs adipose tissue-derived mesenchymal stem cells, BCAA branched chain amino acids, DUX4 double homeobox protein 4, H3K9me3 histone H3K9 trimethylation, KDM4E lysine-specific demethylase 4E, mTORC1 mechanistic target of rapamycin complex 1, S6K1 ribosomal protein S6 kinase polypeptide 1

Next, we sought to identify the mechanisms underlying the upregulation of KDM4E in ADSCs induced by BCAA. A GSEA showed that the mechanistic target of rapamycin complex 1 (mTORC1) signaling was enriched in BCAA-treated ADSCs (Fig. 5a). As expected, BCAA activated mTORC1 signaling in ADSCs in a dose-dependent manner, as evidenced by increased p-mTOR (S2448) and p-S6K1 (T389) levels (Fig. 5b). Adenovirus vectors carrying short hairpin RNA (shRNA) targeting the gene *Rptor* (sh-*Rptor*) were transfected into ADSCs to silence Raptor expression. Raptor is an indispensable protein component of the mTORC1 complex. A western blot analysis showed that silencing Raptor compromised the mTORC1 activation induced by BCAA, as indicated by the decreased levels of S6K1 phosphorylation (Fig. 5c). Intriguingly, the upregulation of KDM4E and the loss of H3K9me3 induced by BCAA were prevented by Raptor silencing (Fig. 5c), revealing the essential role of mTORC1 in BCAA-induced KDM4E upregulation and H3K9me3 loss. Next, the transcription factors regulating Kdm4e were predicted using the methods described, and the results revealed that double homeobox protein 4 (DUX4) ranked the first in the candidates (Fig. 5d). This prediction was consistent with the previous report that *Kdm4e* is a potential target transcriptionally regulated by DUX4.^18^ Notably, a conserved binding motif in the Kdm4e promoter of DUX4 was confirmed (Fig. 5e). BCAA upregulated the expression of DUX4 in ADSCs in a dose-dependent manner (Fig. 5f). Knockdown of DUX4 by siRNA prevented the upregulation of KDM4E and preserved H3K9me3 abundance in BCAA-treated ADSCs (Fig. 5g). In HEK293 cells, DUX4 overexpression increased the mRNA levels of *Kdm4e* in a dose-dependent manner up to approximately 1700-fold (Fig. 5h). To determine whether there is a direct relationship between DUX4 and KDM4E, a plasmid overexpressing DUX4 and a luciferase reporter plasmid carrying the Kdm4e promoter were co-transfected into HEK293 cells. DUX4 increased the luciferase activity of the Kdm4e promoter in a dose-dependent manner (Fig. 5i). Additionally, chromatin immunoprecipitation-quantitative PCR (ChIP-qPCR) revealed that DUX4 directly combined with the promoter of the Kdm4e gene (Fig. 5j). Intriguingly, the direct combination between DUX4 protein and Kdm4e promoter was validated in rat ADSCs, which was further amplified by BCAA challenge (Fig. 5j). These results indicate that DUX4 is a direct transcription factor that regulates *Kdm4e* gene transcription in ADSCs. Moreover, the upregulation of DUX4 induced by BCAA was markedly attenuated by Raptor silencing in ADSCs, suggesting that DUX4 is a downstream target of mTORC1 (Fig. 5k). To observe whether excessive BCAA accumulation regulates KDM4E expression in the post-ischemic heart, the expression of KDM4E was observed in the hearts of the mice from the sham, MI, and MI plus oral BCAA administration group, respectively, at day 7 post-MI, a time point that BCAA peaked in the post-ischemic heart. The results showed that myocardial KDM4E expression was not influenced by MI or MI plus external BCAA supplementation (supplementary Fig. 7). This observation suggest that BCAA-mediated KDM4E upregulation might be specific to the MSCs, likely due to DUX4, the upstream transcription factor of KDM4E, being mainly expressed in embryonic tissues and stem cells, whereas it is restricted to adult tissues and cells.^18^ Collectively, these results show that a novel BCAA/mTORC1/DUX4 axis is critical for the upregulation of KDM4E and the loss of H3K9me3 in ADSCs.

### The BCAA catabolic capability of ADSCs determined their adaptation to the high BCAA levels and cardioreparative efficacy

**Fig. 6:**
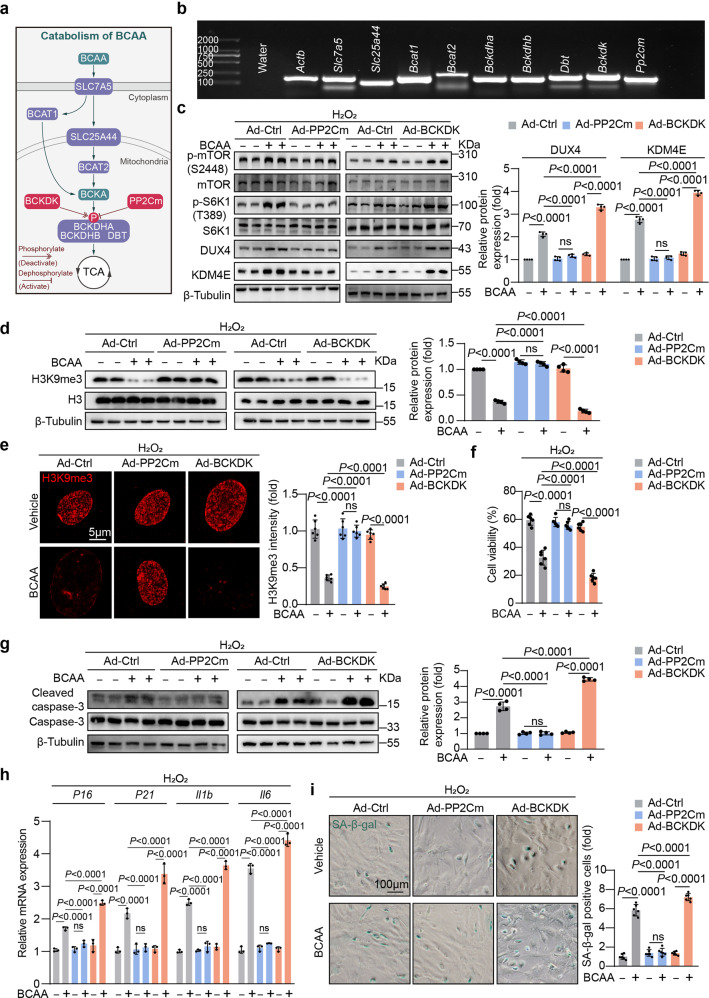
The BCAA catabolic capability of ADSCs determined their adaptation to the extracellular high BCAA milieu. **a** Schematic diagram of genes related to BCAA uptake, transportation, and catabolism. **b** mRNA levels of genes involved in BCAA metabolism measured by RT-PCR. Water was used as the negative control and *Actb* was used as the positive control. **c** ADSCs were transfected with control adenovirus (Ad-Ctrl), adenovirus overexpressing PP2Cm (Ad-PP2Cm) or adenovirus overexpressing BCKDK (Ad-BCKDK) and treated with or without BCAA (3.432 mM) in the presence of hydrogen peroxide (100 μM). Representative blots and quantification of p-mTOR (S2448), mTOR, p-S6K1 (T389), S6K1, DUX4, KDM4E, and β-tubulin as determined by western blot analysis. β-Tubulin was used as the loading control. **d** Representative blots and quantification of H3K9me3, histone H3, and β-tubulin. **e** Representative immunostaining images and quantification of H3K9me3. **f** ADSCs viability was determined by CCK-8 assay. **g** Representative blots of cleaved caspase-3, caspase-3, and β-tubulin. β-Tubulin was used as the loading control. **h** The mRNA levels of *P16*, *P21*, *Il1b*, and *Il6* were analysed by RT-qPCR. **i** ADSC premature senescence was induced as methods described. Representative images and quantification of SA-β-gal positive cells. The data are shown as the means ± SD. The data were analysed by two-way ANOVA followed by Bonferroni post hoc test. ADSCs adipose tissue-derived mesenchymal stem cells, BCAA branched chain amino acids, BCKDK BCKDHA kinase, DUX4 double homeobox protein 4, H3K9me3 histone H3K9 trimethylation, KDM4E lysine-specific demethylase 4E, mTOR the mechanistic target of rapamycin, PP2Cm mitochondrial matrix-targeted protein phosphatase 2C family member, SA-β-gal senescence-associated β-galactosidase, S6K1 ribosomal protein S6 kinase polypeptide 1

We wondered whether the BCAA catabolic capability of ADSCs influences their adaptation to the high BCAA levels in the extracellular milieu. It remains unknown whether BCAA can be catabolized in ADSCs. We measured the expression levels of genes involved in BCAA uptake and catabolism in rodent ADSCs. The results showed that a series of genes critical for BCAA uptake and catabolism (e.g., *Bcat*, *Bckdha*, *Bckdhb*, *Bckdk*, and *Pp2cm*) were abundantly expressed in ADSCs, suggesting that ADSCs can catabolize BCAA (Fig. 6a, b). PP2Cm and BCKDHA kinase (BCKDK) control BCKDHA activity by regulating its phosphorylation at serine 293.^5^ Utilizing adenovirus vectors, we overexpressed PP2Cm in ADSCs to enhance their BCAA catabolic capability or overexpressed BCKDK in ADSCs to suppress their BCAA catabolic capability (supplementary Fig. 8a). As expected, PP2Cm overexpression decreased the phosphorylation of BCKDHA at serine 293, indicating the activation of BCKDHA (supplementary Fig. 8b). In contrast, BCKDK overexpression increased BCKDHA phosphorylation at serine 293, indicating the inactivation of BCKDHA (supplementary Fig. 8b). Notably, PP2Cm overexpression repressed mTORC1/DUX4/KDM4E axis activation, preserving H3K9me3 abundance in ADSCs treated with pathological levels of BCAA (Fig. 6c–e). In contrast, the upregulation of the mTORC1/DUX4/KDM4E axis and the loss of H3K9me3 induced by BCAA were further amplified in BCKDK-overexpressing ADSCs (Fig. 6c–e). Hence, PP2Cm-overexpressing ADSCs were resistant to the adverse impact of BCAA on their survival and senescence, as determined by cleaved caspase-3 expression, cell viability assay, senescence-associated gene expression, and SA-β-gal staining (Fig. 6f–i). Compared with the control ADSCs, BCKDK-overexpressing ADSCs were more vulnerable to death and senescent phenotype acquisition induced by high levels of BCAA (Fig. 6f–i). Together, these in vitro data provide evidence that the BCAA catabolic capability in ADSCs, which is tightly regulated by PP2Cm and BCKDK, determines cell adaptation to high levels of BCAA in the extracellular microenvironment.

**Fig. 7:**
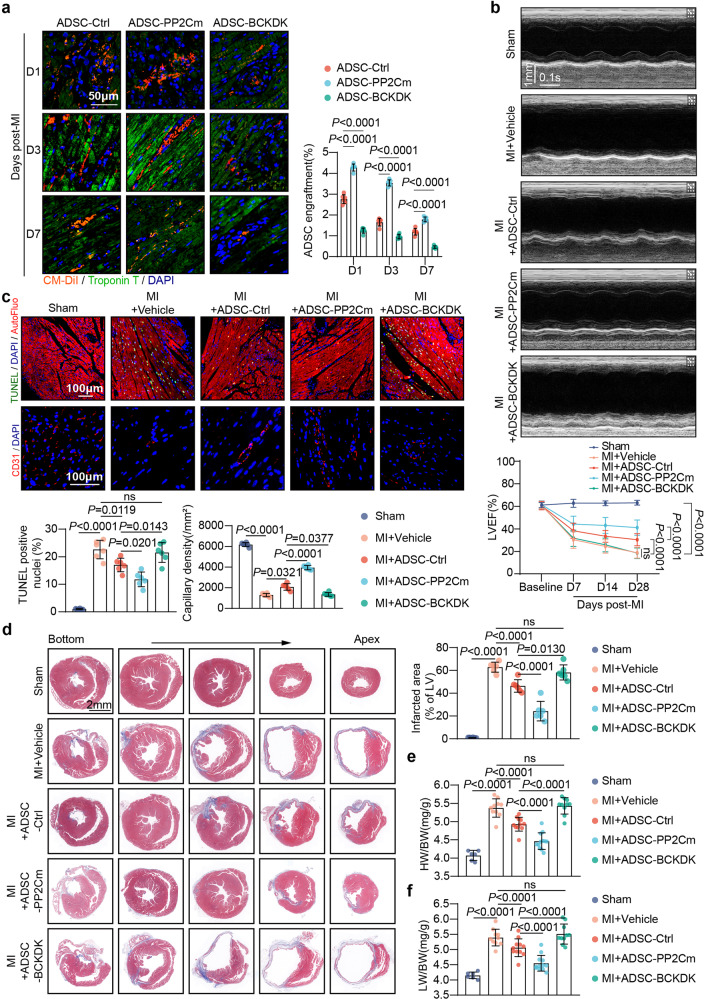
The BCAA catabolic capability of ADSCs determined their adaptation and cardioprotective efficacy in the post-infarction heart. **a** Retention of ADSCs labeled by the cell tracker CM-DiI (red) was visualized by immunostaining of heart tissues 1, 3, and 7 d post-MI. Troponin T staining (green) indicates cardiomyocytes. Left panel, representative images; right panel, ratios of the number of engrafted ADSCs to the number of cardiomyocytes. ADSC-Ctrl ADSCs transfected with adenovirus carrying empty plasmids, ADSC-PP2Cm ADSCs transfected with adenovirus overexpressing PP2Cm, ADSC-BCKDK ADSCs transfected with adenovirus overexpressing BCKDK. **b** Top panel, representative echocardiographic images 28 d post-MI; bottom panel, quantification of LVEF at baseline and 7, 14, and 28 d after MI. **c** Representative images of TUNEL staining (top) and CD31 immunostaining in the infarct border zone 3-d post-MI (middle). Bottom panel, quantification of TUNEL and CD31 staining intensity. **d** Left panel, representative images of Masson’s trichrome stained tissue from the bottom to the apex of hearts 28 d post-MI. right panel, quantification values of infarcted area as determined with ImageJ software. **e** HW/BW ratios 28 d post-MI; **f** LW/BW ratios 28 d post-MI. The data are shown as the means ± SD. Data were analysed by one-way ANOVA followed by a Bonferroni post hoc test. ADSCs adipose tissue-derived mesenchymal stem cells, AutoFluo autofluorescence, BCAA branched-chain amino acids, BCKDK BCKDHA kinase, HW/BW heart weight/body weight ratio, LW/BW lung weight/body weight ratio, MI myocardial infarction, PP2Cm mitochondrial matrix-targeted protein phosphatase 2C family member, TUNEL transferase-mediated dUTP nick-end labeling

We sought to determine whether intervening the BCAA catabolic capability of ADSCs would influence their adaptation to the post-infarction heart milieu and ultimately determine their cardioprotective efficacy. ADSCs transfected with adenovirus vectors carrying an empty control (ADSC-Ctrl), PP2Cm (ADSC-PP2Cm), or BCKDK (ADSC-BCKDK) were separately delivered into the heart immediately after MI. The intramyocardial injection of ADSC-Ctrl, ADSC-PP2Cm, or ADSC-BCKDK had little influence on myocardial BCAA levels at day 7 post-MI, when the BCAA levels in the post-ischemic heart peaked (supplementary Fig. 8d). However, compared with ADSC-Ctrl, ADSC-PP2Cm exhibited a much higher engraftment rate in the myocardial milieu 1, 3, and 7 d post-MI (Fig. 7a). However, the retention rate of ADSC-BCKDK was significantly lower than that of ADSC-Ctrl in the heart 1, 3, and 7 d post-MI (Fig. 7a). These results suggest that the BCAA catabolic capability of ADSCs determines their retention in the post-infarction heart milieu. Importantly, compared with the ADSC-Ctrl mice, the mice that received intramyocardial ADSC-PP2Cm injection showed better ventricular contractile function after MI, as evidenced by consecutive echocardiography, whereas ADSC-BCKDK failed to improve cardiac function following MI (Fig. 7b). In comparison to the effects of ADSC-Ctrl, ADSC-PP2Cm mediated superior pro-survival and pro-angiogenetic effects in the post-infarction heart, as evidenced by lower cardiomyocyte apoptosis and higher capillary density (Fig. 7c). However, ADSC-BCKDK delivered to post-infarction hearts lost their pro-survival and pro-angiogenetic potential (Fig. 7c). Masson’s trichrome staining revealed that the cardiac size and myocardial fibrotic area were slightly reduced by ADSC-Ctrl transplantation but were significantly reduced by ADSC-PP2Cm treatment (Fig. 7d). ADSC-BCKDK exerted little impact on post-MI ventricular enlargement or fibrotic scar expansion compared with the effect of the MI + Vehicle treatment (Fig. 7d). The HW/BW and LW/BW ratios were much lower in mice that received ADSC-PP2Cm treatment than in mice treated with ADSC-Ctrl (Fig. 7e, f). In contrast, the HW/BW and LW/BW ratios were not significantly different in the MI + Vehicle and MI + ADSC-BCKDK groups (Fig. 7e, f). Taken together, these results demonstrate that the BCAA catabolic capability of ADSCs determines their adaptation to high levels of BCAA in the local milieu of the post-infarction heart, thus influencing their cardioprotective efficacy in ischemic heart injury.

## Discussion

Despite being proven that stem cells can not directly differentiate into cardiomyocytes and regenerate the heart, stem cell therapy indeed exerts a consistent improvement in cardiac function in patients and animals with myocardial ischemic injury via incompletely understood mechanisms.^19^ MSCs, mainly originating from bone marrow, adipose tissue, and umbilical cord, continue to attract attention and receive recognition because of the large amount of preclinical evidence supporting their multiple cardioprotective potentials.^20^ Although the mechanisms remain controversial and debates are underway, a large amount of evidence has demonstrated that modifying stem cells by genetic or pharmacological approaches to enhance their adaption to the ischemic heart milieu can improve the survival, engraftment, and cardioprotection of implanted stem cells.^12,21–26^ To date, the poor survival of delivered stem cells and insufficiency of stem cell engraftment in the damaged tissue milieu remain a bottleneck that is challenging in the field.

The study’s most crucial discovery is that abnormal BCAA accumulation is a metabolic change in the post-infarcted heart that prevents implanted MSCs from surviving and engrafting in the post-infarction heart. Metabolic reprogramming is a feature of the failing myocardium and results in significant changes in intramyocardial metabolite levels. However, it is unclear how these metabolic changes affect the fate of MSCs implanted into the heart. In addition to other investigators, we had previously clarified that excessive BCAA accumulation due to impaired myocardial BCAA catabolism represents one of the most significant metabolic changes in the failing heart, determining the progression of adverse cardiac remodeling and heart failure.^6–9^ Here, we observed, for the first time, that the engraftment and cardioprotection of ADSCs were significantly attenuated upon delivery into the post-MI hearts characterized by excessive BCAA accumulation. Furthermore, the deleterious stress-induced damage and senescent phenotypes of ADSCs were considerably amplified in response to BCAA exposure at pathological levels seen in the post-ischemic heart. These in vivo and in vitro results demonstrate that excessive BCAA accumulation in the local milieu is disadvantageous for the retention and cardioprotection of MSCs delivered into the post-infarction heart. These findings, which were acquired for the first time to the best of our knowledge, reveal a previously unappreciated clue that metabolic alterations in the post-ischemic heart are a key factor impacting the fate of implanted MSCs and their therapeutic efficacy.

Second, we assessed the direct influence of BCAA at pathological levels on the fate of ADSCs. We observed that without any detrimental external stress, BCAA at concentrations ranging from 0.432 mM (the physiological level) to 3.432 mM were nontoxic to ADSCs. However, in response to detrimental challenges mimicking the damaged heart milieu (in this case, oxidative stress), BCAA at pathological levels under disease conditions greatly sensitized ADSCs to cell death and senescence. The acquisition of these adverse phenotypes induced by BCAA weakened the paracrine cardioprotective potential of these cells. These results demonstrate that excessive BCAA accumulation in the local milieu is an unfavorable metabolic factor that creates disadvantages for implanted MSCs. Circulating and local BCAA levels are elevated in multiple disease conditions, such as heart failure, type 2 diabetes mellitus, obesity, non-alcoholic fatty liver, chronic kidney diseases, and cancers.^27–31^ These results suggest that the above-mentioned comorbidities in patients may reduce the therapeutic efficacy of MSC-based therapy due to the aberrant accumulation of BCAA.

Third, we clarified a novel metabolite-signaling-epigenetic modification mechanism in the regulation of stress-induced premature senescence. Cellular senescence is regulated by coordinated mechanisms of signal transduction, cellular metabolism, and epigenetic modifications.^32^ However, little is known about how these processes are orchestrated to regulate cellular senescence. Our discovery-driven experiments revealed that BCAA caused a significant loss of H3K9me3 abundance in ADSCs challenged by oxidative stress. H3K9me3 is a common epigenetic histone modification that maintains heterochromatin and nuclear integrity. The loss of H3K9me3 is well recognized as an epigenetic hallmark of the ageing MSCs and this change accelerates the senescent phenotypes in MSCs.^16,17^ The decrease of H3K9me3 drives the disorganization of the heterochromatin, resulting in the detachment of genomic lamina-associated domains from the nuclear lamina, the discontinuous nuclear envelop, increased chromatin accessibility, and aberrant repetitive sequence transcription. These epigenetic changes facilitate the genome injury as well as the senescence and death of MSCs in response to external stresses.^32,33^ In this study, we found that BCAA at pathological levels accelerated the loss of H3K9me3 and heterochromatin in ADSCs, thus sensitizing these cells to stress-induced death and premature senescence. As essential amino acids, BCAA are important metabolites for the activation of mTORC1 signaling, a key nutrient sensor in both invertebrate and vertebrate individuals.^5^ Sustained mTORC1 activation has been identified as a major cause of cellular senescence and individual ageing through elusive mechanisms.^34^ Here we discovered a novel mTORC1/DUX4/KDM4E pathway that was identified as the cause of H3K9me3 loss and the acquisition of adverse phenotypes in BCAA-challenged ADSCs. Disruption of this pathway prevented the H3K9me3 loss and cell injury induced by BCAA. These results demonstrate a previously unrecognized metabolite-signaling-epigenetic modification mechanism involving BCAA, mTORC1, and H3K9me3 in regulating stress-induced cell injury and premature senescence.

Finally, we found that the BCAA catabolic capability of ADSCs determines their adaptation to the local milieu characterized by excessive BCAA accumulation in vitro and in vivo. We identified, for the first time, that the genes involved in BCAA catabolism were highly expressed in ADSCs, suggesting that ADSCs can catabolize BCAA. PP2Cm-overexpressing ADSCs exhibited increased BCKDHA activity, whereas BCKDHA activity was suppressed in ADSCs overexpressing BCKDK. Intriguingly, PP2Cm-overexpressing ADSCs were resistant to the unfavorable effects mediated by BCAA in vitro. In contrast, ADSCs overexpressing BCKDK were more vulnerable than control ADSCs to injuries induced by BCAA. In vivo experiments showed that PP2Cm-overexpressing ADSCs had a much higher retention rate and better cardioprotective effects against post-MI cardiac dysfunction and remodeling, whereas ADSCs with BCKDK overexpression were intolerant of the post-myocardial infarction milieu and completely lost their cardioreparative potential. These results show that the BCAA catabolic capability of ADSCs determines their adaptation to high levels of BCAA in the post-infarction heart milieu and thus determines their cardioprotective efficacy.

**Fig. 8:**
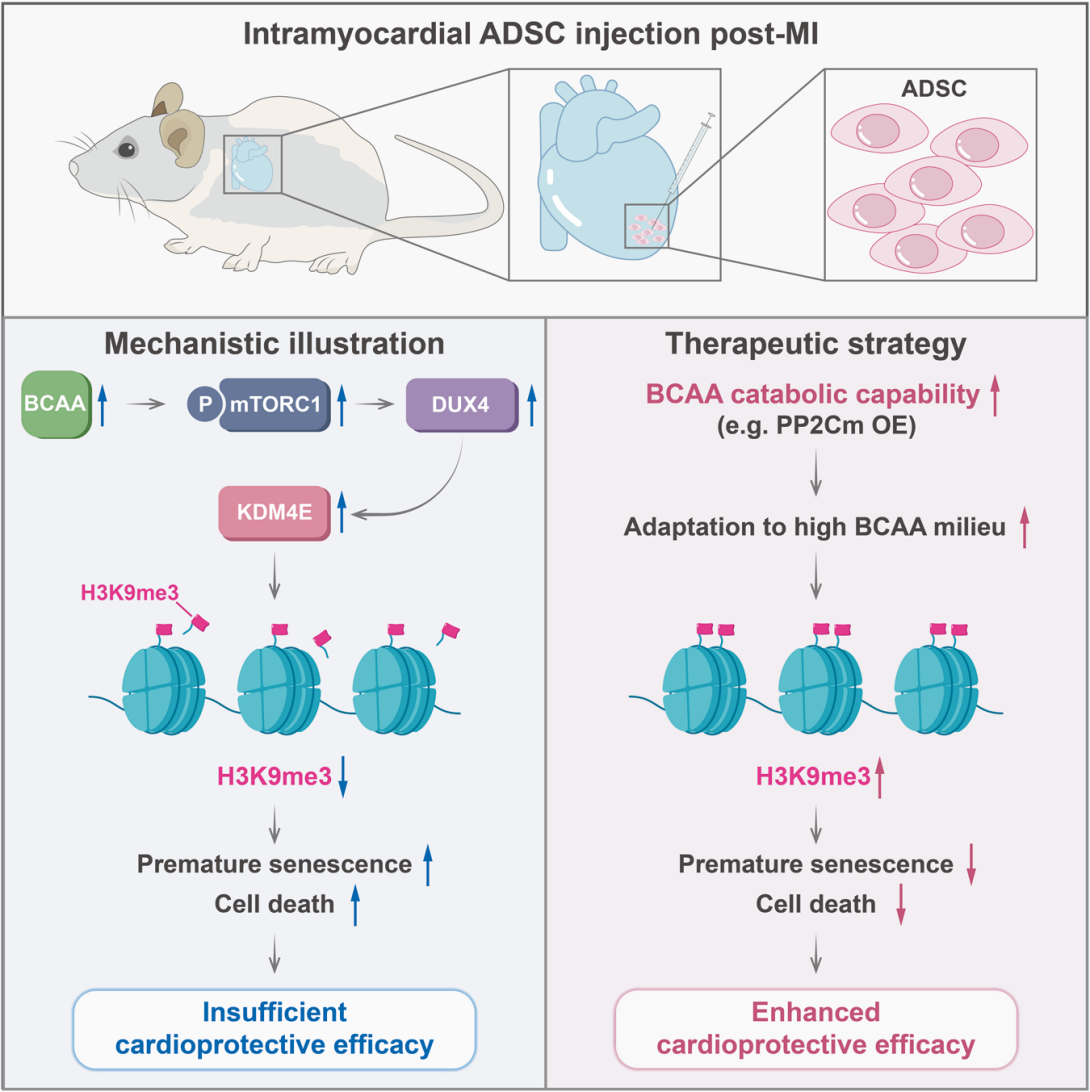
Schematic illustration. After MI, myocardial BCAA catabolism is disrupted, and BCAA are aberrantly accumulated in the post-ischemic heart. Excessive BCAA accumulation in the post-MI heart milieu results in the activation of mTORC1 signaling, the upregulation of DUX4 and KDM4E, and the loss of H3K9me3 in MSCs implanted into the post-ischemic heart. The loss of H3K9me3 drives premature senescence and death of implanted MSCs, thus restricting their cardioprotective effects. Boosting BCAA catabolism in MSCs via appropriate approaches (e.g. PP2Cm overexpression) can improve the adaptation of MSCs to the high BCAA milieu in the post-MI heart, increase the retention of MSCs after the implantation, and strengthen the therapeutic efficacy of MSC-based therapy

In summary, we show that excessive BCAA accumulation, one of the most significant metabolic changes in the post-ischemic heart, is disadvantageous to MSC retention and inhibits their cardioprotective effects. Moreover, we discovered that BCAA, mTORC1, and H3K9me3 comprise a novel metabolite-signaling-epigenetic modification axis that together regulates MSC survival and senescence. From a translational perspective, the present study emphasizes that enhancing BCAA catabolism in MSCs via the appropriate approaches might be a feasible strategy to improve the adaptation of MSCs to the ischemic myocardial milieu and may thus optimize the cardioreparative efficacy of MSC-based therapy (Fig. 8).

## Materials and methods

### Animals, myocardial infarction, and intramyocardial ADSC injection

All animal experimental procedures were approved by the Animal Care and Use Committee of the Fourth Military Medical University and adhered to the National Institutes of Health Guidelines for the Use of Laboratory Animals. Transgenic mice with global PP2Cm knocked out (KO) were obtained and maintained as we previously described.^7^ To establish MI models, mice were anaesthetized through inhalation of isoflurane (1–2%), and left anterior descending coronary artery ligation surgery was performed as we previously described.^6,7^ Intramyocardial adipose tissue-derived mesenchymal stem cells (ADSCs) injection was performed as we previously described.^12^ Rodent ADSCs were isolated from adipose tissues of adult male Sprague–Dawley rats and cultured in vitro. Passage 2 ADSCs were used in the present study. ADSCs (2 × 10^5^) suspended in 20 µl of ice-cold phosphate-buffered saline (PBS, containing 0.2 mM EDTA, pH = 7.3) were carefully intramyocardially injected at three different sites in the peri-infarct zone immediately after ligation of the coronary artery. Sham group mice were subjected to all the same surgical procedures as the experimental mice, but not ligation of the coronary artery. Mice in the MI + vehicle group were injected with only PBS at a volume equal to the treatment injection. A person experienced in surgeries and blinded to the experiment performed the MI operation and intramyocardial ADSC administration. ADSCs were labeled during incubation with the widely used cell membrane dye CM-DiI (Thermo Fisher Scientific, C7001, 5 μM) for 20 min in culture medium before injection, as we previously described.^35^

### Echocardiography

Mice were anaesthetized by inhaling 1–2% isoflurane. M-mode echocardiography was performed with a VisualSonics 770 small animal echo system as we previously described.^6,7^ Hearts were viewed in the short axis between the two papillary muscles. Each measurement was obtained by calculating the average of results obtained from three consecutive heartbeats. Left ventricular ejection fraction (LVEF), LV end-systolic dimension (LVESD), and LV end-diastolic dimension (LVEDD) were automatically calculated by the echocardiography software.

### Measurement of cardiac BCAA levels

Cardiac BCAA concentrations were measured using a BCAA detection kit (BioVision, K564-100, USA) according to the manufacturer’s protocol. The absorbance was measured at 450 nm using an Epoch Microplate Spectrophotometer (BioTek Instruments, USA). The BCAA concentration was calculated based on the standard curve and normalized to the sample protein concentration.

### Rat ADSC isolation and culture

ADSCs were isolated from adult male Sprague–Dawley rats as we previously described.^12^ In brief, epididymal adipose tissue was isolated from rats under full anesthesia. The adipose tissue was rinsed with ice-cold PBS, and the blood vessels were removed, cut into 2 × 2-mm pieces and digested with collagenase I (1 mg/ml, Gibco, 17100017) at 37 °C for 30 min. Tissue lysates were filtered through a 70-μm strainer (Falcon) and centrifuged at 800 g for 5 min. After removing supernatant lipids, the cells were centrifuged at 800 g for another 5 min with Red cell lysis buffer. The cells were then resuspended and cultured in high-glucose Dulbecco’s modified Eagle’s medium (DMEM/HG, HyClone) containing 10% foetal bovine serum (FBS, CellMax) and 1% penicillin/streptomycin (HyClone). After the cells were cultured overnight, the medium was replaced with fresh medium to remove nonadherent cells. Adherent cells were presumed to be ADSC. Passage 2 ADSCs were used in all experiments. For experiments related to BCAA, customized BCAA-free DMEM/HG (Boster, China) supplemented with exogenous BCAA (weight ratio of leucine:valine:isoleucine = 2:1:1; L8912, V0513, and I7403, respectively; Sigma-Aldrich) was used to exclude the impact of endogenous BCAA contained in regular culture medium.^7,8^

### NRVM isolation

Neonatal rat ventricular myocytes (NRVMs) were isolated from 1- to 2-d-old Sprague–Dawley pups as we described previously.^7^ Hearts were immediately removed under adequate anesthesia, and ventricles were carefully minced. After rinsing with PBS, ventricles were digested with 1 mg/ml collagenase I (Gibco, 17100017). After filtration through a 100-μm strainer (Falcon) and centrifugation at 800 g for 5 min, the cells were resuspended in DMEM/HG (HyClone) containing 10% FBS (CellMax), 10 mM HEPES, 0.1 mM BrdU (Sigma-Aldrich), and 1% penicillin/streptomycin (HyClone) and plated for 90 min to allow the attachment of fast-adhering fibroblasts. Nonadherent NRVMs were resuspended and seeded in plates with culture medium lacking BrdU.

### In vitro hypoxia/reoxygenation model

H/R NRVM models were established as we previously described.^7,36^ The model NRVMs were subjected to 9 h of hypoxia in a hypoxic chamber with 95% N_2_ and 5% CO_2_ at 37 °C followed by 3 h of reoxygenation. NRVM viability was evaluated with the cell viability assay described herein.

### Cell viability assay

Cell viability was assessed by cell counting kit-8 (CCK-8) assay (TargetMol, C0005). ADSCs (2 × 103) were seeded in wells in 96-well plates. The cells were treated with 100 μM hydrogen peroxide (Sigma-Aldrich, 323381) or PBS with various concentrations of BCAA for 12 h and then incubated with CCK-8 (10 μl per well) for another 2 h at 37 °C. The absorbance was measured at 450 nm with an Epoch Microplate Spectrophotometer (BioTek Instruments, USA), and the viability was determined with the following equation: %ADSC viability = (absorbance of cells treated with hydrogen peroxide/absorbance of cells treated with PBS) × 100%. For the viability assay of NRVMs, 2 × 10^3^ NRVMs per well were incubated with CCK-8 for 2 h at 37 °C after H/R induction, and viability was determined with the following equation: %NRVM viability = (absorbance of NRVM with H/R/absorbance of NRVM without H/R) × 100%.

### Western blot analysis

Tissues or cells were lysed using RIPA lysis buffer (Beyotime, China) supplemented with 1× protease inhibitor cocktail (TargetMol, C0001) and 1× phosphatase inhibitor cocktail (TargetMol, C0004). A BCA kit (Thermo Fisher Scientific) was used to measure the protein concentration. Twenty to sixty micrograms of total protein per sample was separated in SDS-PAGE gels and transferred to PVDF membranes (Millipore); the membranes were blocked with fast-blocking buffer (NCM Biotech, Suzhou, China) for 10 min. The membranes were then incubated overnight with primary antibodies at 4 °C and subsequently with HRP-conjugated secondary antibodies (1:5000, CoWin Biosciences, China) at room temperature for 1 h. Blots were scanned with a ChemiDocTM MP Imaging System (Bio-Rad) and analysed with Image Lab 4.0 software (Bio-Rad). Detailed information on the primary antibodies is presented in supplementary Table 1.

### Real-time quantitative PCR

Total RNA was extracted from cells using TRIzol reagent (Invitrogen) and reverse transcribed to cDNA using the PrimeScriptTM RT reagent kit with gDNA Eraser (TaKaRa, RR047A). Real-time quantitative PCR (RT-qPCR) was performed using TB Green Premix Ex TaqTM II (TaKaRa, RR820A) with a StepOnePlus real-time PCR system (Applied Biosystems). The primer sequences are presented in supplementary Table 2.

### Immunofluorescence staining

Cells were fixed with 4% paraformaldehyde for 20 min and permeabilized with 0.4% Triton 100×-100 (Beyotime, China) in PBS for 10 min at room temperature. After rinsing with PBS, the cells were blocked with 2.5% bovine serum albumin (BSA) in PBS for 1 h at room temperature and incubated overnight with primary antibodies at 4 °C. The cells were stained with secondary antibodies for 1 h and then with 4’,6-diamidino-2-phenylindole (DAPI, Beyotime) stain for 5 min at room temperature. Images were acquired with a confocal microscope (LSM880, Carl Zeiss, Germany) using identical exposure parameters. Negative controls stained with only secondary antibodies were included in all experiments. Images were analysed with ImageJ software. Fixed tissues were sectioned at a thickness of 5 μm following a series of deparaffinization and dehydration steps. Slides were then subjected to antigen retrieval in hot citric acid buffer. After cooling, the slides were permeabilized, blocked, incubated with primary antibodies, incubated with secondary antibodies, and stained with DAPI as previously described. Images were scanned with a Pannoramic MIDI scanner (3D HISTECH), viewed with CaseViewer software (3D HISTECH), and analysed with ImageJ software. The primary antibodies are listed in supplementary Table 1.

### Three-dimensional (3D) Z-stack image reconstruction

Three-dimensional Z-stack image reconstruction was generated as described previously with minor modifications.^37^ Images were captured with a confocal microscope (LSM880, Carl Zeiss, Germany) in ‘Z-Stack’ mode. All images were acquired in 8-bit format at 1024×1024 pixel resolution. The images were then converted using an Imaris File Converter (9.6.0, Bitplane Scientific Solutions) and reconstructed with Imaris software (7.4.2, Bitplane Scientific Solutions). The ‘Surface’ tool was used to generate 3D signals in each channel. Using these rendered surfaces, the spatial localization of H3K9me3 and Lamin A/C was visualized.

### Masson’s trichrome staining

Fixed whole-heart samples were cut into five 5-μm-thick sections from the bottom to the apex of each heart. Slides were stained with Masson’s trichrome according to the manufacturer’s protocol (Sigma-Aldrich, HT15). Images were scanned by a Pannoramic MIDI scanner (3D HISTECH) and analysed with CaseViewer software (3D HISTECH). The infarcted area was identified as the average ratio of the fibrosis area to the total left ventricle area.

### ADSC premature senescence induction

ADSCs treated with different concentrations of BCAA were stressed with 100 μM hydrogen peroxide for 1 d, and then, the medium was replaced with hydrogen peroxide-free medium containing different concentrations of BCAA and incubated for another 2 d to induce ADSC premature senescence.

### Senescence-associated β-galactosidase staining

SA-β-gal staining was performed according to the manufacturer’s protocol (Cell Signaling Technology, #9860). Briefly, cells were fixed with 1× fixative solution for 10 min at room temperature and incubated overnight with β-galactosidase staining solution (pH = 6.0) at 37 °C. Bright-field images were obtained with an Olympus microscope system (Olympus, IX71, Japan). The SA-β-gal-positive cell percentage was calculated with ImageJ software.

### TUNEL staining

TdT-mediated dUTP nick-end labeling (TUNEL) staining was performed using a one-step TUNEL apoptosis assay kit (Beyotime, C1090) according to the manufacturer’s protocol. Images were captured using an Olympus microscope system (Olympus, IX71, Japan). Positive cell percentages were analysed with ImageJ software.

### RNA sequencing

ADSCs with or without BCAA (3.432 mM) were incubated for 24 h in the presence of hydrogen perperoxide (100 μM) and then used for RNA-seq (*n* = 3 per group). RNA library preparation, sequencing, and analysis were performed by Jiayin Biotechnology Ltd. (Shanghai, China). In brief, RNA was isolated using TRIzol reagent (Invitrogen). A total of 3 μg of RNA per sample was utilized for RNA library preparation with the NEBNext® UltraTM RNA Library Prep Kit for Illumina® (NEB, USA). The library was sequenced on an Illumina NovaSeq6000 platform. After quality control, reads were aligned to the reference genome using the STAR read mapper.^38^ Then, HTSeq v0.6.0 software was used to count the read numbers mapped to each gene, and the fragments per kilobase of transcript sequence per million (FPKM) data were used for further analysis. Differentially expressed genes (DEGs) were identified using the DESeq2 package (|fold change | ≥1.2, *P* < 0.05). Gene Ontology (GO) enrichment analysis was performed and the results visualized with the OmicShare tool, an online platform for data analysis (https://www.omicshare.com/tools/Home/Soft/enrich_circle). Gene set enrichment analysis (GSEA) was performed with GSEA 4.1.0 software (Broad Institute, Cambridge, MA). The raw RNA-sequencing data are available at the GEO database at the accession number GSE183028.

The mouse MI RNA-seq data (GSE95755) and human ischemic cardiomyopathy (ICM) RNA-seq data (GSE48166) were obtained from the Gene Expression Omnibus (GEO) database. DEGs were identified with DESeq2 (mouse MI:|fold change| ≥ 1.5, *P* < 0.05; human ICM:|fold change| ≥ 1.2, *P* < 0.05). Kyoto Encyclopedia of Genes and Genomes (KEGG) enrichment analysis was performed using the OmicShare tool (https://www.omicshare.com/tools/home/report/koenrich.html).

### Cleavage under targets and tagmentation

CUT&Tag of ADSCs treated with or without 3.432 mM BCAA for 24 h in the presence of hydrogen perperoxide (100 μM) was performed by Jiayin Biotechnology Ltd. (Shanghai, China) as described previously with minor modifications.^39^ A total of 5 × 105 nuclei per sample were gently washed twice with wash buffer (20 mM HEPES, pH 7.5; 150 mM NaCl; 0.5 mM spermidine; and 1× protease inhibitor cocktail). Concanavalin A-coated magnetic beads (10 μl, Bangs Laboratories) were added to each sample and incubated for 10 min at RT. After removal of unbound cells in the supernatant and resuspension of bead-bound cells with DIG wash buffer (20 mM HEPES, pH 7.5; 150 mM NaCl; 0.5 mM spermidine; 1× protease inhibitor cocktail; 0.05% digitonin; and 2 mM EDTA), cells were incubated overnight with an anti-H3K9me3 antibody (1:50, ABclonal, A2360) or rabbit normal IgG (1:50, Millipore, 12–370) at 4 °C with rotation. Then, the primary antibody was removed using a magnet stand. Secondary antibody (1:100, anti-rabbit IgG antibody, Millipore, AP132) was diluted in DIG wash buffer, and cells were incubated at room temperature for 60 min. After washing with DIG wash buffer and subjecting the cells on beads to the magnet stand 3 times, the cells were incubated with a pA-Tn5 adapter complex (1:100, diluted in Dig-med buffer containing 0.01% digitonin; 20 mM HEPES, pH 7.5; 300 mM NaCl; 0.5 mM spermidine; and 1× protease inhibitor cocktail) at room temperature for 1 h. Cells were then washed 3 times in Dig-med buffer, resuspended in tagmentation buffer (10 mM MgCl2 in DIG-med buffer), and incubated at 37 °C for 1 h. DNA was purified using an phenol-chloroform-isoamyl alcohol extraction and ethanol precipitation. Purified DNA was then used to amplify the libraries. The size distribution of the libraries was determined by Agilent 4200 TapeStation analysis. Sequencing was performed using a 150 bp paired-end sequence and an Illumina NovaSeq 6000 sequencer. After quality control, the reads were mapped to the reference genome using the BWA program. Peak calling was performed using MACS2 software with a cut-off q value < 0.05. Peaks were annotated with the annotate peak functions with the ChIPseeker package. Differential peaks were assessed with DESeq2 (|fold change|≥2, *P* < 0.05). H3K9me3 signals in chromosomes were visualized using RIdeograms. The whole CUT&Tag data are available in the GEO database at the accession number GSE183028.

### Adenovirus transfection

Adenovirus vectors carrying plasmids overexpressing PP2Cm (Ad-PP2Cm) constructed by Hanbio Co., Ltd. (Shanghai, China) or BCKDK (Ad-BCKDK) constructed by GeneChem Co., Ltd. (Shanghai, China) were used to transfect ADSCs that had reached 60% confluence. Specifically, Ad-PP2Cm, Ad-BCKDK, or Ad-Ctrl, an adenovirus containing empty plasmids, was transfected into ADSCs for 8 h at a multiplicity of infection (MOI) of 100. The medium was changed to fresh medium for another 48 h before use in experiments.

### RNA interference and transfection

Small interfering RNAs (siRNAs) targeting the Kdm4e (si-Kdm4e), Dux4 (si-Dux4), and scramble siRNA (si-NC) genes were constructed by Tsingke Biotechnology Co., Ltd. (Beijing, China). Cells grown to 60% confluence were transfected with these siRNAs using Xfect RNA transfection reagent (TaKaRa, 631450) according to the manufacturer’s protocol. The final concentrations of siRNAs was 100 pM. Adenoviruses carrying short hairpin RNAs targeting the Rptor gene (sh-Rptor#1 and sh-Rptor#2) or a scramble RNA (sh-NC) were constructed by Hanbio Co., Ltd. (Shanghai, China). Cells at 60% confluence were transfected with sh-Rptor#1, sh-Rptor#2, or sh-NC for 8 h at an MOI of 50. The medium was removed and fresh medium added, and the cells were then cultured for 48 h before use in experiments. The siRNA and shRNA sequences are listed in supplementary Table 3.

### Dual-luciferase activity assay

PGL4.10-Kdm4e, pEGFP-C1-Dux4, PGL4.70-Renilla, and empty control plasmids were constructed by Tsingke Biotechnology Co., Ltd. (Beijing, China). Firefly luciferase expression was under the control of the Kdm4e promoter. HEK293 cells were plated into 24-well plates and co-transfected with plasmids using Xfect™ transfection reagent following the manufacturer’s instructions (TaKaRa, 631717). After transfection, a dual-luciferase activity assay was performed using a dual luciferase assay kit (Promega, E1910) per the manufacturer’s instructions.

### Chromatin immunoprecipitation-qPCR

ChIP-qPCR was performed following the manufacturer’s protocol (Cell Signaling Technology, 9003 s). In brief, 4 × 106 HEK293 cells per sample were cross-linked with formaldehyde, and then, the cells were digested and centrifuged. The supernatants were incubated overnight with mouse IgG (1:100, Santa Cruz, sc-69786) or anti-DUX4 antibody (1:100, Santa Cruz, sc-376490) at 4 °C with rotation. Subsequently, the supernatants were incubated with protein G, washed several times, and subjected to magnetic separation. After removing the supernatant, the cells were eluted and reverse cross-linked. DNA was then purified in spin columns. The purified DNA was then subjected to RT-qPCR to determine the occupancy of DUX4 at the Kdm4e promoter region. The following primers were used for ChIP-qPCR: human Kdm4e promoter: forward (5′-3′), CAGCTGTCCCCACAGTTGAT; reverse (5′-3′), TCTCCGCATTCACCCACATTAT. Rat Kdm4e promoter: forward (5′-3′), CTATTGCAGAGGAGTGCCCG; reverse (5′-3′), ATGGATCATCCTCGCGTCTC.

### Flow cytometry analysis of ADSC apoptosis

Flow cytometry analysis to determine the rate of apoptotic ADSCs was performed using a FITC Annexin V apoptosis detection kit following the manufacturer’s instructions (BD Pharmingen, 556547). All measurements were obtained with an Epics XL-MCL flow cytometer (Beckman Coulter), and the data were analysed using FlowJo v10 software. The apoptotic cells were counted as PI-negative and Annexin V-positive (early apoptotic) or PI-positive and Annexin V-positive (late apoptotic) cells.

### Statistical analysis

All data are presented as the means ± SD. The data were analysed with GraphPad Prism 8 software (GraphPad Software Inc.). The differences between two groups were compared by two-tailed unpaired Student’s *t* test. For comparisons of more than two groups, one-way or two-way ANOVA followed by Bonferroni post hoc test was performed. The Kaplan–Meier survival curves were analysed by Gehan-Breslow-Wilcoxon test. A *P* value < 0.05 was considered statistically significant.

## Supplementary information

Supplementary figures and tables

## Data Availability

Cardiac transcriptome data of human and murine ischemic cardiomyopathy were obtained from the GEO database. The GEO accession numbers are GSE95755 and GSE48166. The raw RNA-sequencing and CUT & Tag data generated in this study have been deposited at the GEO database. The GEO accession number is GSE183028. Any additional information required to reanalyse the data reported in this paper is available from the lead contact upon request.
