# Supplementary material for: Excessive branched-chain amino acid accumulation restricts mesenchymal stem cell-based therapy efficacy in myocardial infarction
Source: Signal Transduct Target Ther. 2022 Jun 3;7:171. doi: 10.1038/s41392-022-00971-7 (PMC9163108; doi:10.1038/s41392-022-00971-7)
Supplement: Supplementary file 1 — Supplementary figures and tables [file 41392_2022_971_MOESM1_ESM.docx]

Supplementary Materials for

Excessive branched-chain amino acid accumulation restricts mesenchymal stem cell-based therapy efficacy in myocardial infarction

Fuyang Zhang1#*, Guangyu Hu1#, Xiyao Chen2#, Ling Zhang1, Lanyan Guo1, Congye Li1, Hang Zhao1, Zhe Cui1, Xiong Guo1, Fangfang Sun1, Dandan Song1, Wenjun Yan1, Yunlong Xia1, Shan Wang1, Miaomiao Fan1, Ling Tao1*

1. Department of Cardiology, 2. Department of Geriatrics, Xijing Hospital, the Fourth Military Medical University, Xi'an 710032, China.

Correspondence to: lingtao@fmmu.edu.cn (to TL) or plazhangfuyang@163.com (to ZFY).

**This PDF file includes:**

Figures. S1 to S8

Tables S1 to S3


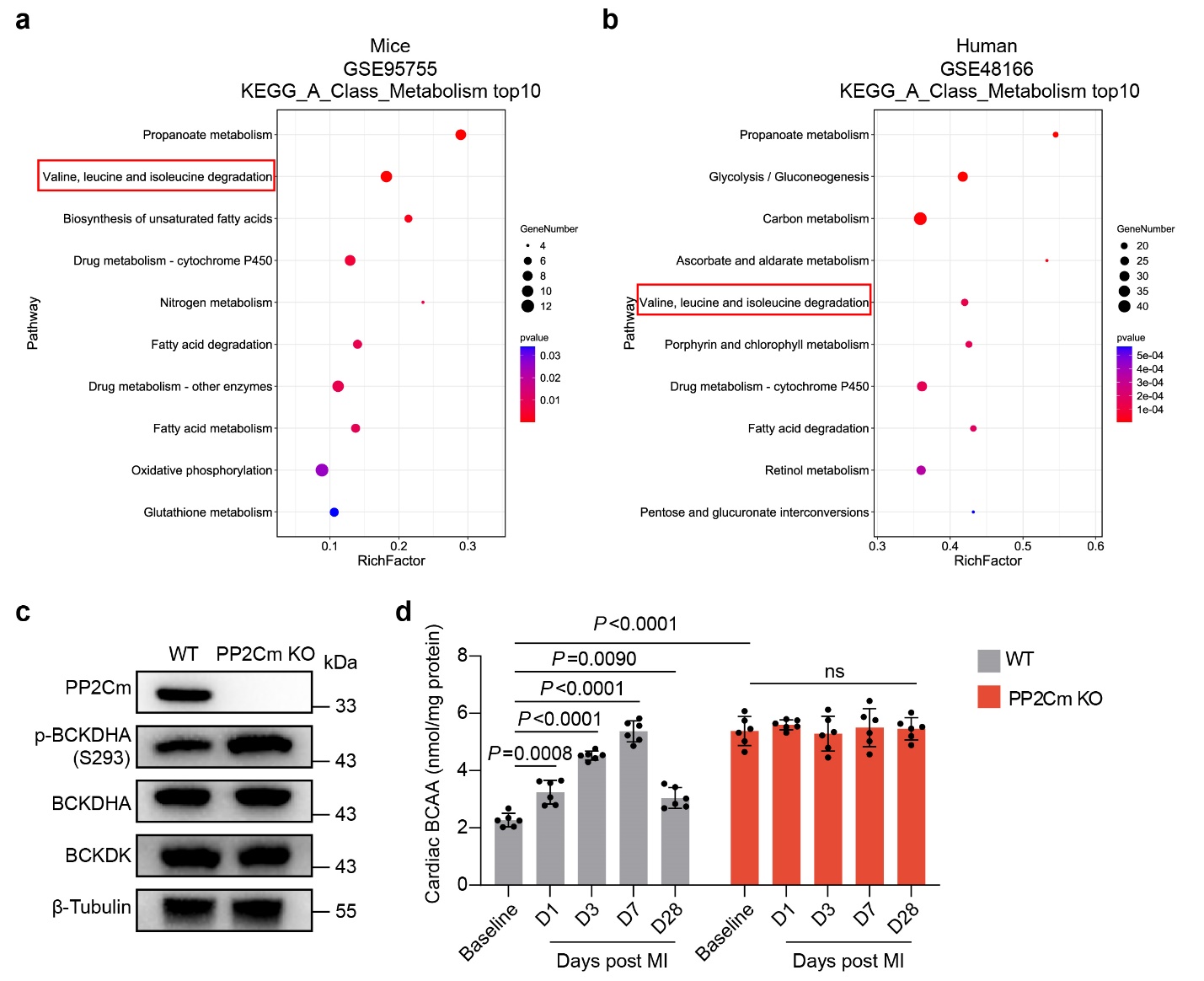


Figure. S1. BCAA catabolic defects were seen in the post-ischemic heart.

(a) KEGG analysis of DEGs downregulated in the hearts of mice with ICM compared to healthy controls. (b) KEGG analysis of DEGs downregulated in hearts of humans with ICM compared to healthy donors. (c) Total proteins were isolated from the hearts of WT or PP2Cm KO mice. Representative blots of PP2Cm, p-BCKDHA (S293), BCKDHA, BCKDK, and β-tubulin are shown. β-Tubulin was used as the loading control. (d) Cardiac BCAA levels were determined in WT and PP2Cm KO mice at baseline and 1, 3, 7, and 28 d post-MI. The data are shown as the means ± SD. The data shown in (d) were analysed by 2-way ANOVA followed by Bonferroni post hoc test. BCAA, branched chain amino acids; BCKDHA, branched chain keto acid dehydrogenase E1 subunit α; BCKDK, BCKDHA kinase; DEG, differentially expressed genes; ICM, ischemic cardiomyopathy; KEGG, Kyoto Encyclopaedia of Genes and Genomes; KO, knockout; MI, myocardial infarction; PP2Cm, mitochondrial matrix-targeted protein phosphatase 2C family member; and WT, wild type.


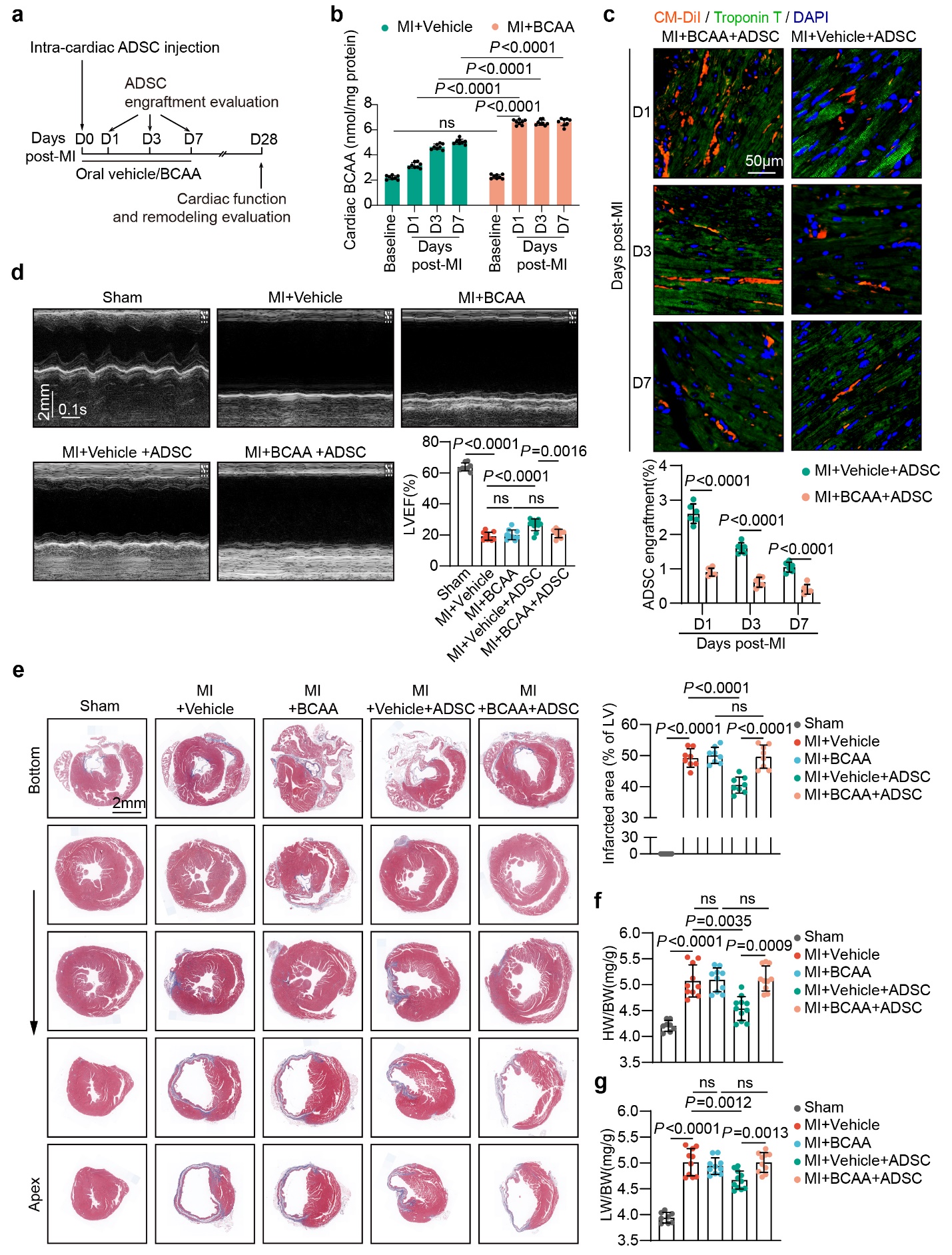


Figure. S2. Exacerbation of myocardial BCAA accumulation weakened implanted ADSC retention and cardioprotection in the post-ischemic heart.

(a) Schematic illustration of the experiment. (b) Cardiac BCAA levels at baseline or at 1, 3, 7 d post-MI. (c) Top panel, representative images of CM-DiI-labelled ADSCs (red) in the peri-infarct region 1, 3, 7 d post-MI. Troponin T staining (green) indicates cardiomyocytes. Bottom panel, number of engrafted ADSCs normalized to the number of cardiomyocytes. (d) Representative echocardiographic images taken 28 d post-MI and LVEF were calculated. (e) Left panel, representative images of Masson’s trichrome staining from the bottom to the apex of hearts 28 d post-MI. Right panel, quantification values of infarcted area. (f) HW/BW ratios 28 d post-MI. (g) LW/BW ratios 28 d post-MI. The data are shown as the means ± SD. The engraftment rates of the ADSCs as determined by unpaired Student’s t tests. Other data were analysed by 1-way ANOVAs with repeated measures followed by Bonferroni post hoc test. ADSCs, adipose tissue-derived mesenchymal stem cells; BCAA, branched chain amino acids; HW/BW, heart weight/ body weight; and LW/BW, lung weight/ body weight.


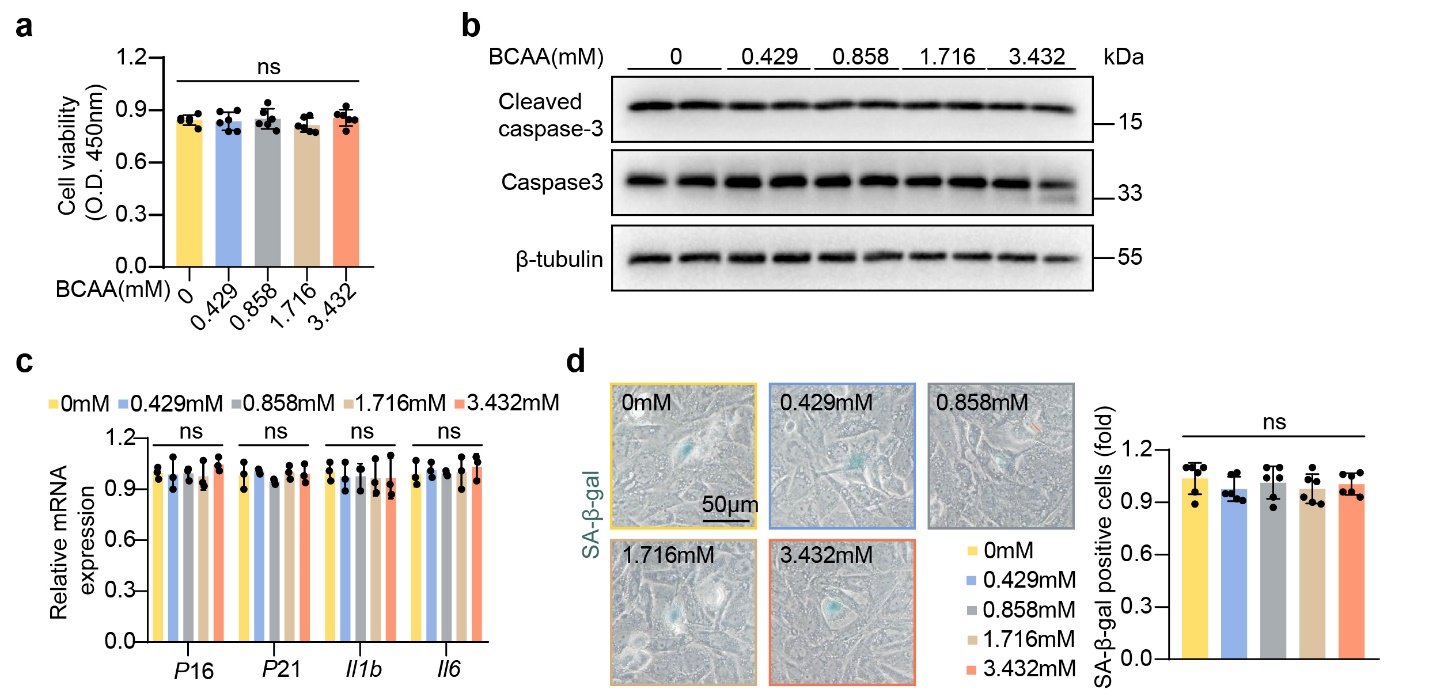


Figure. S3. BCAA were nontoxic to ADSCs in absence of external stress.

(a) Cell viability was measured by CCK-8 assay after cells were treated with different doses of BCAA for 24 h without exposure to external stress. (b) Cleaved caspase-3, caspase-3, and β-actin levels in ADSCs treated with increasing doses of BCAA. β-Actin was used as the loading control. (c) mRNA levels of p16, p21, Il1b, and Il6 as measured by RT-PCR. Actb was used as the loading control. (d) Representative images and quantification of SA-β-gal staining intensity in ADSCs treated with different doses of BCAA for 48 h without exposure to external stress. The data are shown as the means ± SD. The data shown in (a), (c), and (d) were analyzed by 1-way ANOVA followed by Bonferroni post hoc test. Actb, β-actin; ADSCs, adipose tissue-derived mesenchymal stem cells; BCAA, branched chain amino acids; CCK-8, Cell Count Kit-8; Il1b, interleukin-1β; Il6, interleukin-6; and SA-β-gal, senescence-associated β-galactosidase.


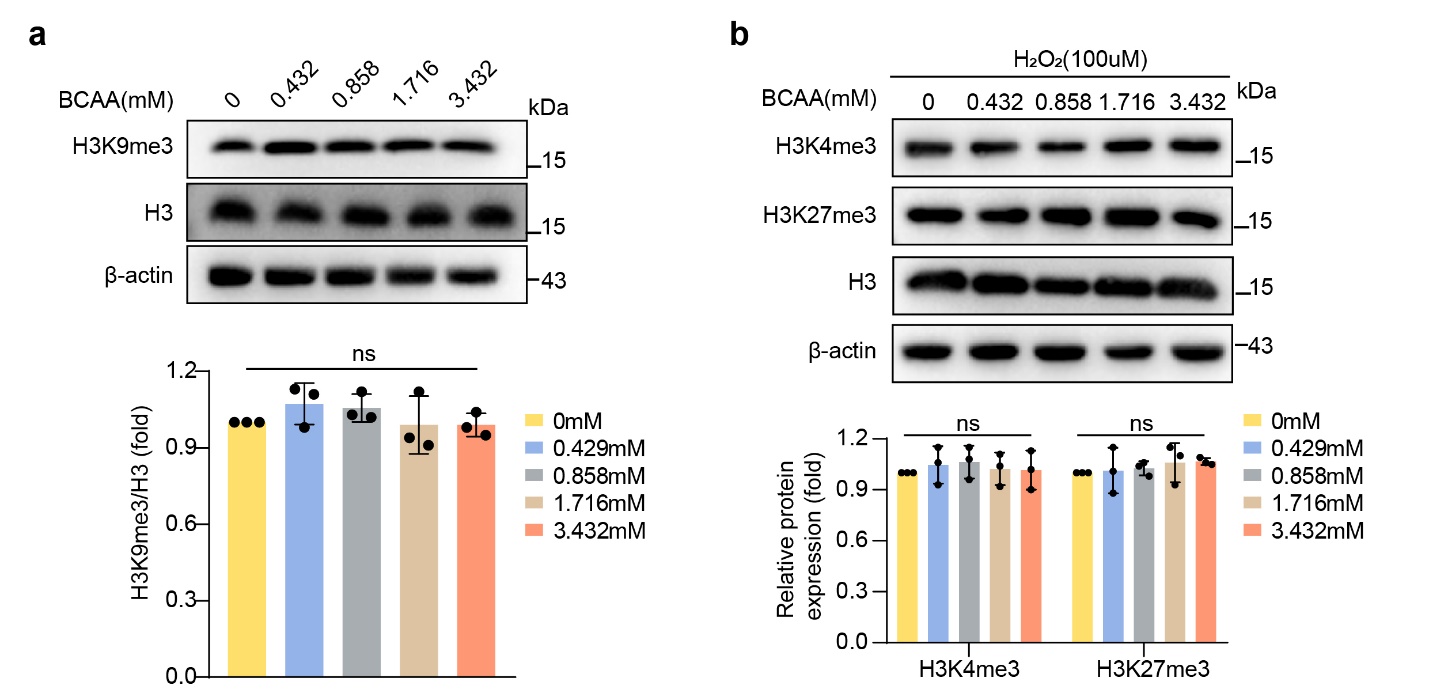


Figure. S4. Effect of BCAA on H3K9me3, H3K4me3, and H3K27me3 in ADSCs.

(a) ADSCs were treated with increasing doses of BCAA for 48 h without exposure to external stress. Representative blots and quantification of H3K9me3, histone H3, and β-actin are shown. (b) ADSCs were treated with increasing doses of BCAA under hydrogen peroxide (100 μM)-induced premature senescence as methods described. Representative blots and quantification of H3K4me3, H3K27me3, histone H3, and β-actin are shown. The data are shown as the means ± SD and were analyzed by 1-way ANOVA followed by Bonferroni post hoc test. ADSCs, adipose tissue-derived mesenchymal stem cells; BCAA, branched chain amino acids; H3K4me3, histone H3K4 trimethylation; H3K27me3, histone H3K27 trimethylation; and H3K9me3; histone H3K9 trimethylation.


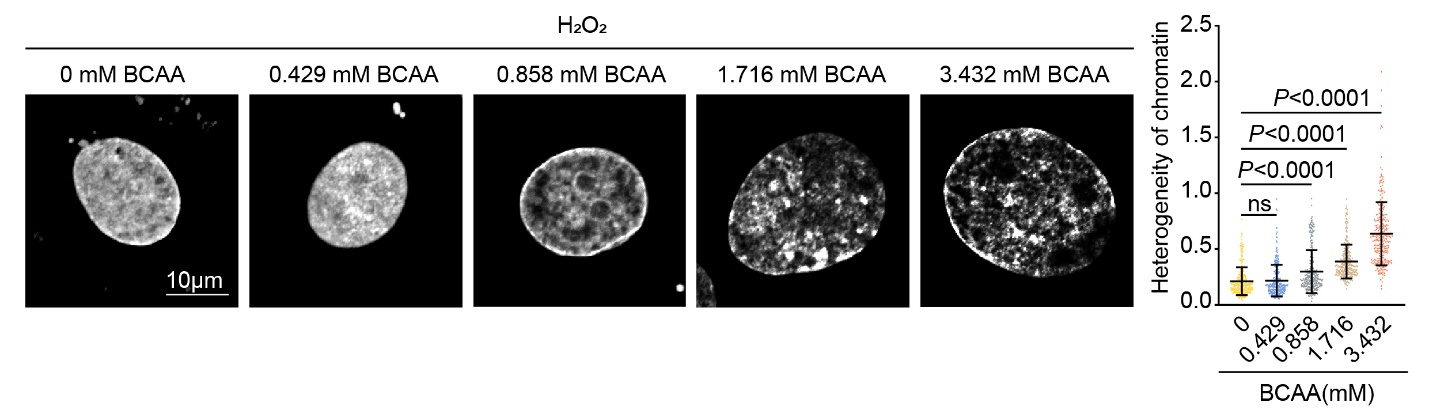


Figure. S5. BCAA accelerated the loss of heterochromatin in ADSCs during stress-induced premature senescence.

ADSCs were treated with increasing doses of BCAA under hydrogen peroxide (100 μM)-induced premature senescence as methods described, and chromatin structures were visualized by DAPI staining. The heterogeneity of the chromatin was determined by coefficient of variation values (pixel-to-pixel variation) of the DAPI intensity. The data are shown as the means ± SD and were analysed by 1-way ANOVA followed by Bonferroni post hoc test. ADSCs, adipose tissue-derived mesenchymal stem cells; BCAA, branched chain amino acids; and DPAI, 4',6-diamidino-2-phenylindole.


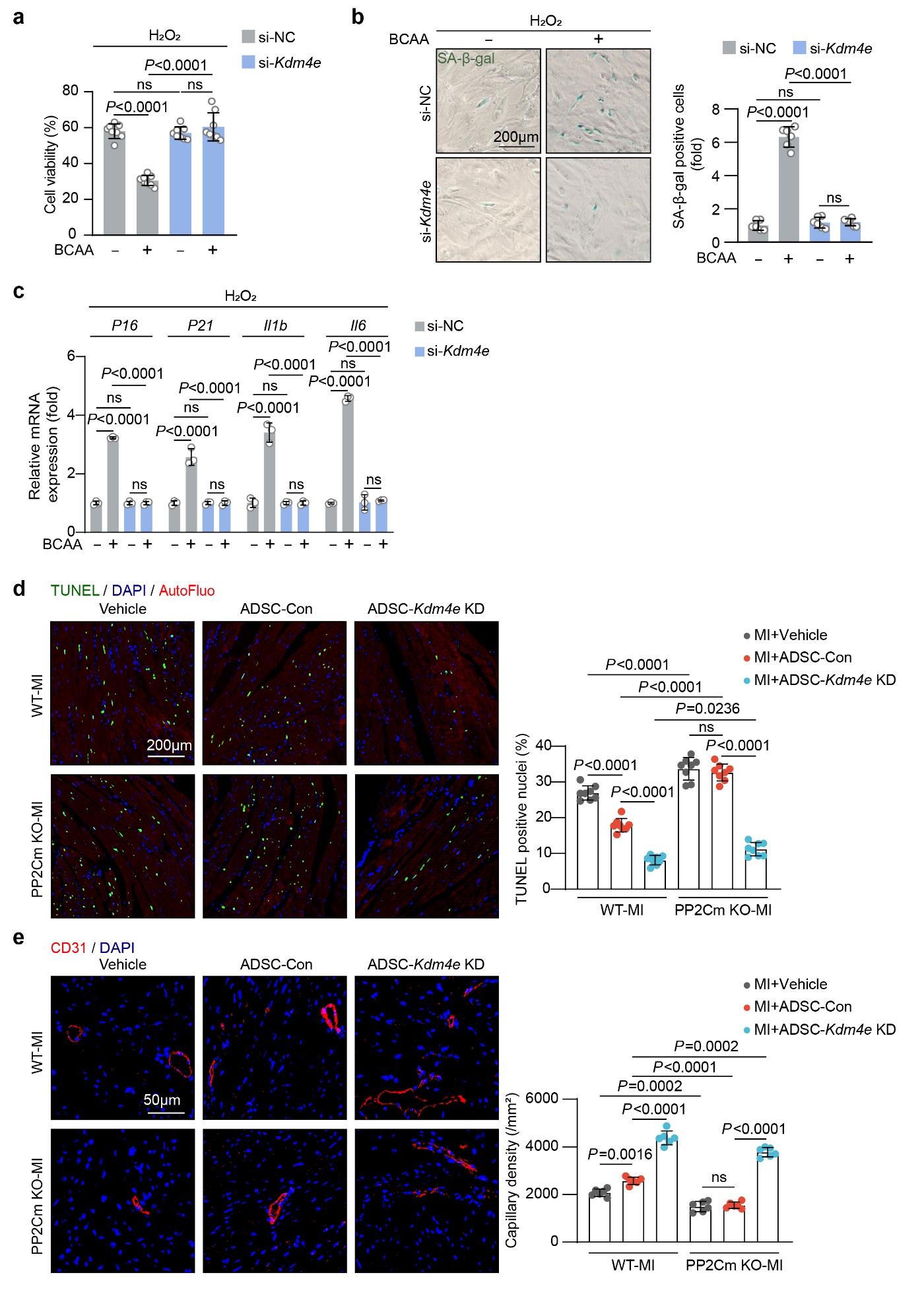


Figure. S6. Silencing of KDM4E ameliorated cell death and senescence and preserved the cardioprotective potential of ADSCs upon exposure to BCAA.

(a) Viability of si-NC- or si-Kdm4e-transfected ADSCs treated with or without BCAA for 24 h in the presence of hydrogen peroxide (100 μM). (b) Representative and quantification of SA-β-gal positive cells in ADSCs transfected with si-NC or si-Kdm4e and treated with or without BCAA (3.432 mM) under hydrogen peroxide (100 μM)-induced premature senescence as methods described. (c) The mRNA levels of P16, P21, Il1b, and Il6 were analysed by RT-qPCR. Actb was used as the endogenous control gene. (d) Representative images and quantification of TUNEL stained cells in the peri-infarction zone 3 d post-MI. (e) Representative images and quantification of CD31 immunostaining in the peri-infarction zone 3 d post-MI. The data are shown as the means ± SD. The data shown in (a), (b), and (c) were analysed by 2-way ANOVA followed by a Bonferroni post hoc test. The data shown in (d) and (e) were analysed by 1-way ANOVAs with repeated measures followed by Bonferroni post hoc test. ADSCs, adipose tissue-derived mesenchymal stem cells; BCAA, branched chain amino acids; DUX4, double homeobox protein 4; H3K9me3, histone H3K9 trimethylation; Il1b, interleukin-1β; Il6, interleukin-6; and KDM4E, lysine-specific demethylase 4E.
TUNEL, transferase-mediated dUTP nick-end labelling.


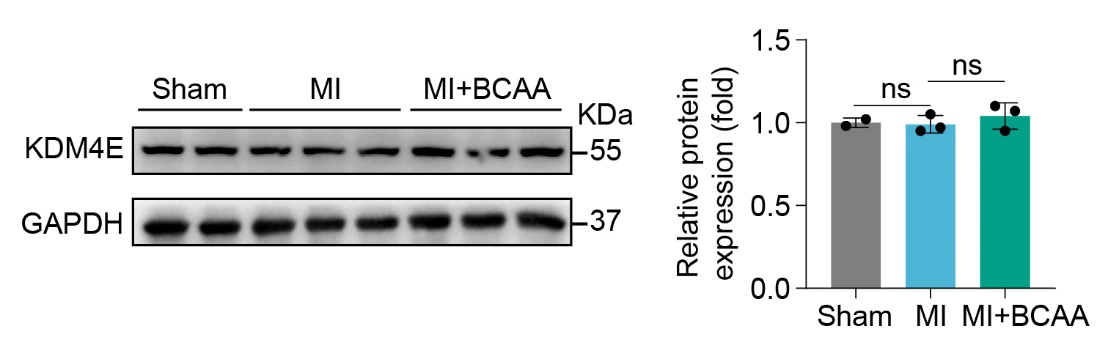


Figure. S7. Myocardial KDM4E expression was not influenced by MI or MI plus oral BCAA supplementation.

The expression of KDM4E in the heart tissues of the mice from Sham, MI, and MI plus oral BCAA supplementation, respectively, was determined by Western blot at day 7 post-MI. The data are shown as the means ± SD and were analysed by 1-way ANOVA followed by Bonferroni post hoc test. MI, myocardial infarction; BCAA, branched chain amino acids; KDM4E, lysine-specific demethylase 4E.


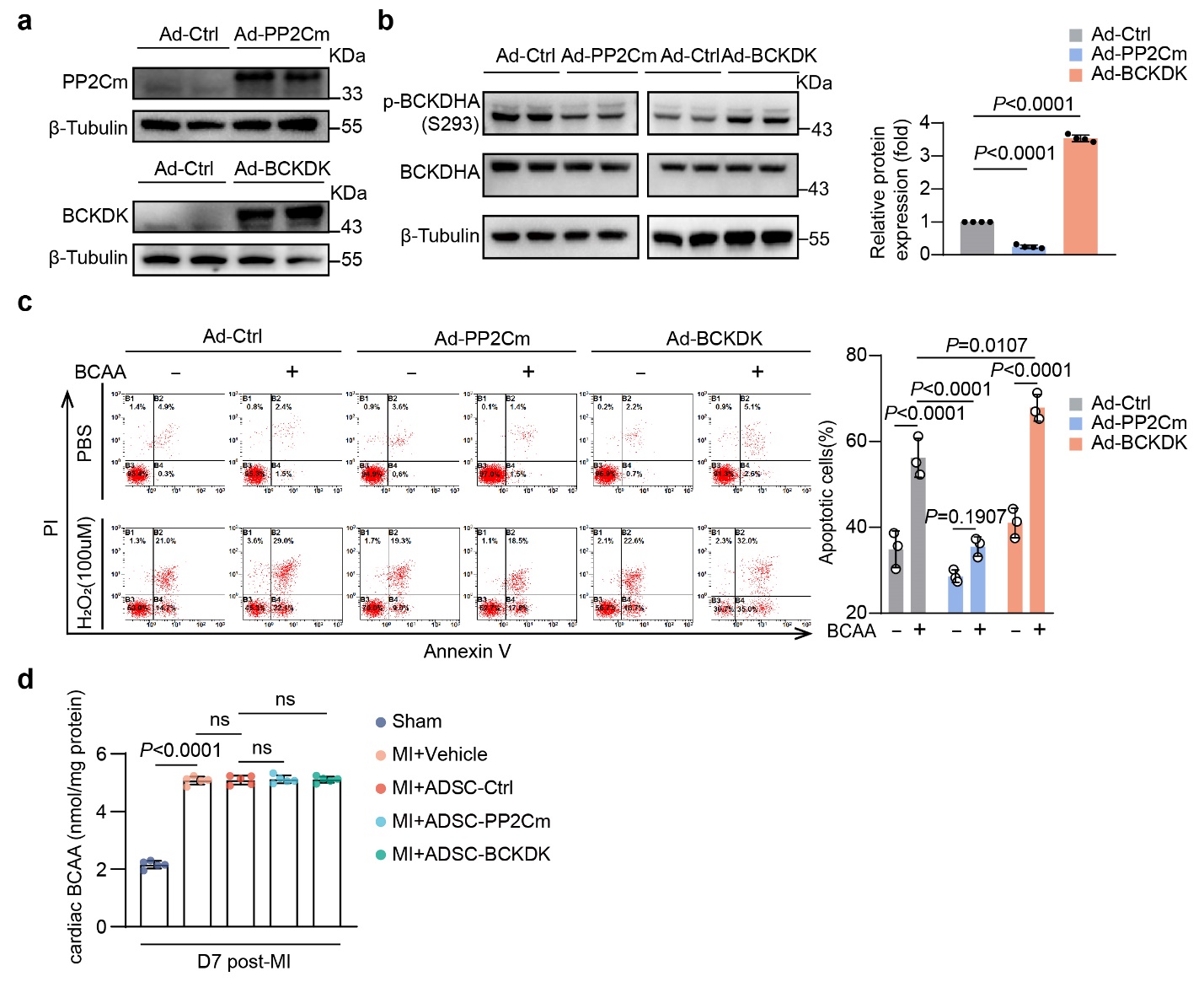


Figure. S8. The BCAA catabolic capability of ADSCs determined their adaptation to the extracellular BCAA milieu.

(a) ADSCs were transfected with adenovirus vectors overexpressing PP2Cm (Ad-PP2Cm), BCKDK (Ad-BCKDK) or an empty control (Ad-Ctrl). Representative blots of PP2Cm, BCKDK, and β-tubulin are shown. β-Tubulin was used as the loading control. (b) In ADSCs transfected with Ad-Ctrl, Ad-PP2Cm or Ad-BCKDK, the p-BCKDHA (S293), BCKDHA, and β-tubulin levels were determined by western blot analysis, and representative blots are shown. (c) ADSCs were transfected with Ad-Ctrl, Ad-PP2Cm or Ad-BCKDK and were treated with or without BCAA (3.432 mM) in the presence of hydrogen peroxide (100 μM) for 24 h. ADSCs apoptosis was assessed by flow cytometry. (d) Cardiac BCAA levels were measured in the mice received ADSC-Ctrl, ADSC-PP2Cm or ADSC-BCKDK treatment, respectively, at day 7 post-MI. The data are shown as the means ± SD. The data shown in (b) were analysed by 1-way ANOVA followed by a Bonferroni post hoc test. The data presented in (c) were analysed by 2-way ANOVA followed by Bonferroni post hoc test. ADSCs, adipose tissue-derived mesenchymal stem cells; BCAA, branched chain amino acids; BCKDHA, branched chain keto acid dehydrogenase E1 subunit α; BCKDK, BCKDHA kinase; and PP2Cm, mitochondrial matrix-targeted protein phosphatase 2C family member.

Table S1.

**Primary antibody information**

| **Target** | **Supplier** | **Catalog** | **Usage** | **Dilution** |
| --- | --- | --- | --- | --- |
| Cleaved-caspase 3 | Immunoway | YM3431 | WB | 1:500 |
| Caspase 3 | Abcam | ab184787 | WB | 1:1000 |
| β-tubulin | Immunoway | YT4780 | WB | 1:2000 |
| P16 | Immunoway | YM0494 | WB | 1:1000 |
| P21 | Immunoway | YM3453 | WB | 1:1000 |
| β-Actin | Immunoway | YM3028 | WB | 1:2000 |
| H3K9me3 | Abclonal | A2360 | WB, IF | 1:2000 (WB), 1:200 (IF) |
| Histone H3 | Abclonal | A2348 | WB | 1:2000 |
| p-mTOR (S2448) | CST | #5536S | WB | 1:1000 |
| mTOR | CST | ab134903 | WB | 1:1000 |
| p-S6K1 (T389) | Abclonal | AP0564 | WB | 1:1000 |
| S6K1 | Abclonal | A2190 | WB | 1:1000 |
| RAPTOR | Abclonal | A8992 | WB | 1:1000 |
| KDM4E | Novus Biologicals | nbp-85142 | WB | 1:1000 |
| DUX4 | Abcam | ab124699 | WB | 1:1000 |
| PP2CM | Proteintech | 14573-1-AP | WB | 1:1000 |
| BCKDK | Abclonal | A8184 | WB | 1:1000 |
| p-BCKDHA (S293) | CST | 40368S | WB | 1:1000 |
| BCKDHA | Abclonal | A9806 | WB | 1:1000 |
| Lamin A/C | Santa Cruz | sc-376248 | IF | 1:200 |
| Troponin T | Servicebio | GB11364 | IF | 1:200 |
| CD31 | Servicebio | GB11063-3 | IF | 1:200 |

Table S2.

**Primer sequences**

| **Genes** | **Forward (5'-3')** | **Reverse (5'-3')** | **Organism** |
| --- | --- | --- | --- |
| *Slc25a44* | CTGGTAGCAAGCGGTGGATG | GTGAGTGGTGGTGGCATTTAC | Rat |
| *Slc7a5* | TGAACTCAGGTCCCCAGACA | AAATGACTGTGGCTGTGGCT | Rat |
| *Bcat1* | AGTGGGGCGAGTTTAAGGTG | CCATGGTTGGAATGTGCAGC | Rat |
| *Bcat2* | TGACGTTAGGGAGGTTGTGC | TCTCTTGAACACCAGGACGC | Rat |
| *Bckdha* | CAACGATGTGTTTGCGGTGT | TTGACCTCATCCACCGAACG | Rat |
| *Bckdhb* | TGCTATGATGCCCTTCGCAA | GTCAGCCAGTATCCGCCATA | Rat |
| *Bckdk* | TCCGAATGCTGGCTACTCAC | AGCGGCATAGGAATGAAGGG | Rat |
| *Pp2cm* | GATCGTCAGCTCAGAGTCGG | AATACTACTGGGTGTGCGGC | Rat |
| *Dbt* | GCTCTTCCCCGATTTGACCA | TCAAGGATGTCTGGTTCGCC | Rat |
| *Actb* | ATCATTGCTCCTCCTGAGCG | CGCAGCTCAGTAACAGTCCG | Rat |
| *P16* | ATCTACTCTCCTCCGCTGGG | TAGTCTCGCGTTGCCAGAAG | Rat |
| *P21* | GACCTAAGCGTACCGTCCAG | CCTGTGTACCCGTTCCCTTC | Rat |
| *Il6* | GCAAGAGACTTCCAGCCAGT | TCTGACAGTGCATCATCGCT | Rat |
| *Il1b* | CAGAACATAAGCCAACAAGTGGT | GATTCTTCCCCTTGAGGCCC | Rat |
| *Ash1l* | AAAGAAAGCCCCACGTCCTC | AACGTAGACATCAACATGAATGGG | Rat |
| *Prdm2* | CGGATTGGTGTCTGGGCTAC | GCATCAATGCACATCCACCC | Rat |
| *Suv39h1* | CTGTGGAGAAAGATGGCGGA | AAACACCAAGGGCAGGACAA | Rat |
| *Suv39h2* | CTCTGGGGTTAGATGGGGCT | CTGGAGCAGTGAGCTTAGGAA | Rat |
| *Setdb1* | CATGCGCCGTGTATCTGGTT | ATGGGGCTGAGGAGAGAAGA | Rat |
| *Setdb2* | TACCTCCTGAGGACCACCAG | CCTGTCAGGTATGGAGCCTG | Rat |
| *Kdm4a* | AGAAAGACAGTGGGATCGGC | TCTTGCGGAACTCACGAACA | Rat |
| *Kdm4b* | CGTAGAGACCAGGCGAAGATAG | TGGAGCTACACCTGTGAGAAG | Rat |
| *Kdm4c* | AAAGCTCCCAAGAGTTTGCAG | TTCTGAAGATGCTCCTATGTTTGCC | Rat |
| *Kdm4d* | AGGCGCAAATAAGTACGGGG | GCGTGTGATGGGACTAGCTT | Rat |
| *Kdm4e* | AGGAGAGCTTGCCAACTGAC | GGGAAGGCTGGTGGAAAGAG | Rat |
| *Kdm4e* | TCCCCAGAACACGAGTCATAC | TTGGGTGGAATTACCTTGGCA | Human |
| *Dux4* | CCATGCCGACTGTTTGCTC | GGTTTGGAACCTGGCAAGG | Human |
| *P16* | CCAACGCACCGAATAGTTACG | TTCCCCTGCAAACTTCGTCC | Human |
| *P21* | CGATGGAACTTCGACTTTGTCA | GCACAAGGGTACAAGACAGTG | Human |
| *Il1b* | ATGATGGCTTATTACAGTGGCAA | GTCGGAGATTCGTAGCTGGA | Human |
| *Il6* | ACTCACCTCTTCAGAACGAATTG | CCATCTTTGGAAGGTTCAGGTTG | Human |
| *Actb* | CTCCATCCTGGCCTCGCTGT | GCTGTCACCTTCACCGTTCC | Human |

Table S3.

**siRNA and shRNA sequences**

| **Target** | **Forward (5’-3’) or top strand** | **Reverse (5’-3’) or bottom strand** |
| --- | --- | --- |
| si-*Kdm4e* | CAAUUCAAGACCUAUUGGATT | UCCAAUAGGUCUUGAAUUGTT |
| si-*Dux4* | GAUUGAUAUUCUAAUCGAATT | UUCGAUUAGAAUAUCAAUCTT |
| si-NC | UUCUCCGAACGUGUCACGUTT | ACGUGACACGUUCGGAGAATT |
| sh-*Rptor*#1 | TCGAGGCTACAGAAGCAGTATGAGATTCAAGAGATCTCATACTGCTTCTGTAGTTTTTTA | AGCTTAAAAAACTACAGAAGCAGTATGAGATCTCTTGAATCTCATACTGCTTCTGTAGCC |
| sh-*Rptor*#2 | TCGAGGGAATCATGAGGTCGTATAATTCAAGAGATTATACGACCTCATGATTCTTTTTTA | AGCTTAAAAAAGAATCATGAGGTCGTATAATCTCTTGAATTATACGACCTCATGATTCC |
| sh-NC | GATCCGTTCTCCGAACGTGTCACGTAATTCAAGAGATTACGTGACACGTTCGGAGAATTTTTTC | AATTGAAAAAATTCTCCGAACGTGTCACGTAATCTCTTGAATTACGTGACACGTTCGGAGAACG |
